# pdCSM-cancer: Using Graph-Based Signatures to Identify Small Molecules with Anticancer Properties

**DOI:** 10.1021/acs.jcim.1c00168

**Published:** 2021-07-02

**Authors:** Raghad Al-Jarf, Alex G. C. de Sá, Douglas E. V. Pires, David B. Ascher

**Affiliations:** † Structural Biology and Bioinformatics, Department of Biochemistry, University of Melbourne, Parkville 3052, Victoria, Australia; ‡ Systems and Computational Biology, Bio21 Institute, University of Melbourne, Parkville 3052, Victoria, Australia; § Computational Biology and Clinical Informatics, Baker Heart and Diabetes Institute, Melbourne 3004, Victoria, Australia; ∥ Baker Department of Cardiometabolic Health, Melbourne Medical School, University of Melbourne, Parkville 3010, Victoria, Australia; ⊥ School of Computing and Information Systems, University of Melbourne, Parkville 3052, Victoria, Australia; # Department of Biochemistry, University of Cambridge, 80 Tennis Ct Rd, Cambridge CB2 1GA, United Kingdom

## Abstract

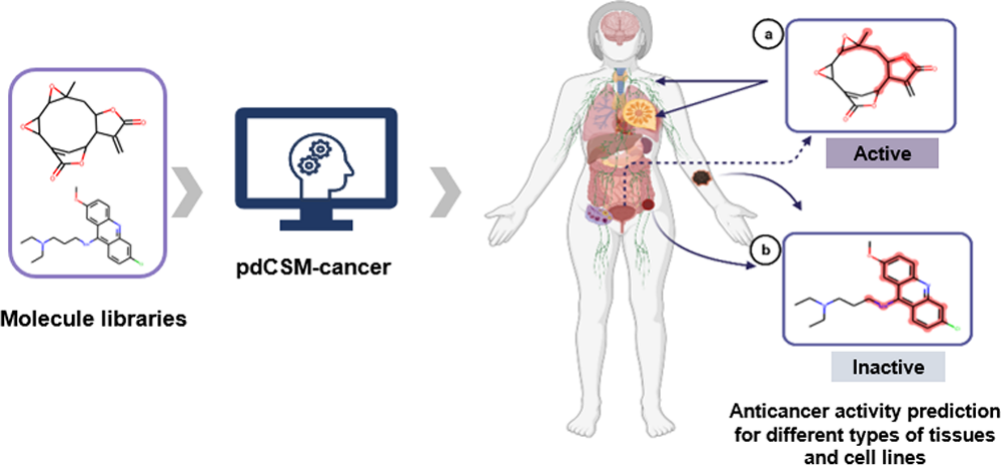

The development of new, effective, and safe drugs to treat cancer remains a challenging and time-consuming task due to limited hit rates, restraining subsequent development efforts. Despite the impressive progress of quantitative structure–activity relationship and machine learning-based models that have been developed to predict molecule pharmacodynamics and bioactivity, they have had mixed success at identifying compounds with anticancer properties against multiple cell lines. Here, we have developed a novel predictive tool, pdCSM-cancer, which uses a graph-based signature representation of the chemical structure of a small molecule in order to accurately predict molecules likely to be active against one or multiple cancer cell lines. pdCSM-cancer represents the most comprehensive anticancer bioactivity prediction platform developed till date, comprising trained and validated models on experimental data of the growth inhibition concentration (GI50%) effects, including over 18,000 compounds, on 9 tumor types and 74 distinct cancer cell lines. Across 10-fold cross-validation, it achieved Pearson’s correlation coefficients of up to 0.74 and comparable performance of up to 0.67 across independent, non-redundant blind tests. Leveraging the insights from these cell line-specific models, we developed a generic predictive model to identify molecules active in at least 60 cell lines. Our final model achieved an area under the receiver operating characteristic curve (AUC) of up to 0.94 on 10-fold cross-validation and up to 0.94 on independent non-redundant blind tests, outperforming alternative approaches. We believe that our predictive tool will provide a valuable resource to optimizing and enriching screening libraries for the identification of effective and safe anticancer molecules. To provide a simple and integrated platform to rapidly screen for potential biologically active molecules with favorable anticancer properties, we made pdCSM-cancer freely available online at http://biosig.unimelb.edu.au/pdcsm_cancer.

## Introduction

Cancer is the second leading cause of death globally, responsible for almost 10 million deaths yearly, according to the World Health Organization.^1^ Despite the evolution of cancer chemotherapy for different tumor types,^2,3^ most existing drugs have many limitations, including undesirable side effects, the lack of efficacy, toxicity, and resistance against current cancer therapy.^4,5^ This has driven the widespread and continuous search for new effective anticancer treatments while remaining a significant challenge to overcome.^6^

One limitation of current screening strategies has been the inefficiencies of current phenotypic and high-throughput screening approaches, which only screen a limited portion of available chemical space and have low hit rates of 0.01–1%, and the size of the molecules screened is a constraint, which may limit or complicate subsequent optimization efforts in terms of both pharmacokinetic and pharmacodynamic properties.^7−9^ Optimizing and enriching these libraries for compounds more likely to demonstrate anticancer potential, and promising pharmacokinetics, could significantly reduce the associated time and development costs. Toward this, several studies have applied statistical and machine learning approaches in order to identify potential anticancer molecules.^5,10,11^ This has been greatly aided by the Development Therapeutics Program (DTP), which experimentally evaluated thousands of small molecules tested against NCI-60 human cancer cell lines.^12^ For example, leveraging this data, Li and Huang used machine learning to identifying molecules with anticancer activity,^10^ and Kumar and colleagues had some success developing quantitative structure–activity relationship (QSAR) models against 16 pancreatic cancer cell lines, regardless of the biological targets of the drugs.^13^ One of their limitations, however, was that no information was provided regarding the physicochemical properties and structural features that a molecule should have to enhance the bioactivity and cytotoxicity against various pancreatic cell lines.

**Figure 1:**
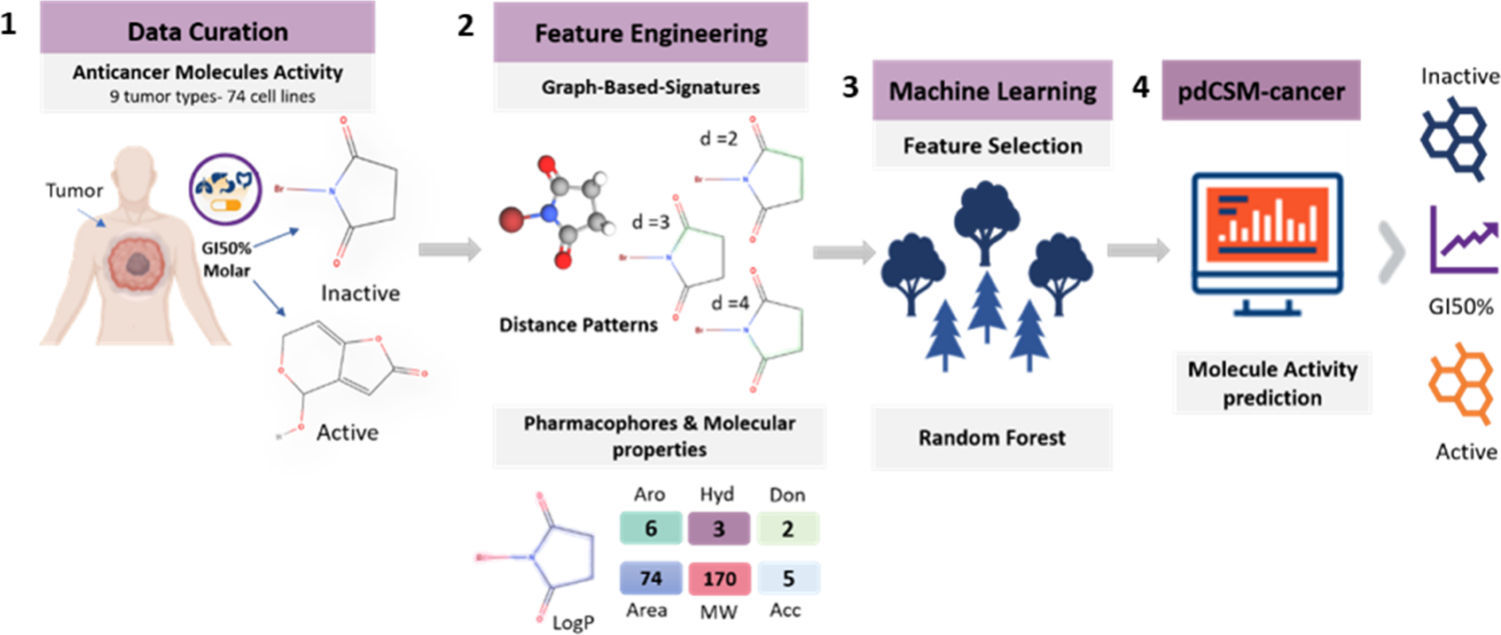
pdCSM-cancer workflow. The developed approach has four major stages. (1) In data curation, small-molecule activity data (in terms of GI50%) were obtained from DTP of NCI^23^ for nine different tumor types (74 cancer cell lines); (2) in feature engineering, two types of features were calculated: (i) graph-based signatures, which represent the chemical geometry and physicochemical properties of small molecules, and (ii) compound general properties and pharmacophores; (3) these features were then employed to train and test predictive models using supervised learning, and model optimization was carried out, via greedy feature selection; (4) finally, the models with the best performance were made available through a user-friendly web interface.

Previous studies have shown that applying the graph-based signature approach is an efficient way to describe chemical, biomolecular data sets and design chemical molecules. In addition, graph-based signatures have been currently used to represent three-dimensional (3D) space of chemical entities as well as to accurately predict their pharmacokinetics, toxicity, and bioactivity properties.^14−22^ Using this concept, we have developed a new machine learning tool, pdCSM-cancer (Figure 1), which can accurately predict small molecules that are likely to be active against one or multiple cancer types over different cell lines.

## Results and Discussion

### Quantitative Correlation of Molecular Properties with Biological Activity

A large and diverse data set of the experimental activity of thousands of molecules against the National Cancer Institute (NCI) 60 cancer cell lines was collected from the literature. Figure S1 represents the distribution of physicochemical properties for compounds with anticancer activity. Most of the active and inactive molecules obeyed Lipinski’s rule of five,^24^ indicating a bias in the primary screening libraries for anticancer drug discovery.

To explore what makes a good hit while screening for anticancer compounds, we assessed whether there was an association between physicochemical properties and biological activity. We observed that the active molecules tended to have a slightly larger topological polar surface area (TPSA), more rings, and a slightly larger number of hydrogen bond acceptors and donors (using a two-sample Kolmogorov–Smirnov test, *p*-value < 2.2e-16) compared to the inactive molecules (Figure S1). This may represent a significant increase in the molecular complexity needed for optimizing potency and safety while designing anticancer molecules.

### Molecular Substructure Mining

In order to better understand and interpret what makes up a molecule with anticancer properties, we investigated common substructures enriched in compounds with anticancer activity using MoSS.^25^ We explored substructure enrichment in a focused group comprising active molecules, in comparison to a complementary set (inactive molecules). In addition, the common substructure enrichment was examined in the NCI-60 panels of cell lines represented as nine distinct tumor types: leukemia, central nervous system (CNS), renal, melanoma, colon, ovarian, breast, lung, and prostate cancers.

**Figure 2:**
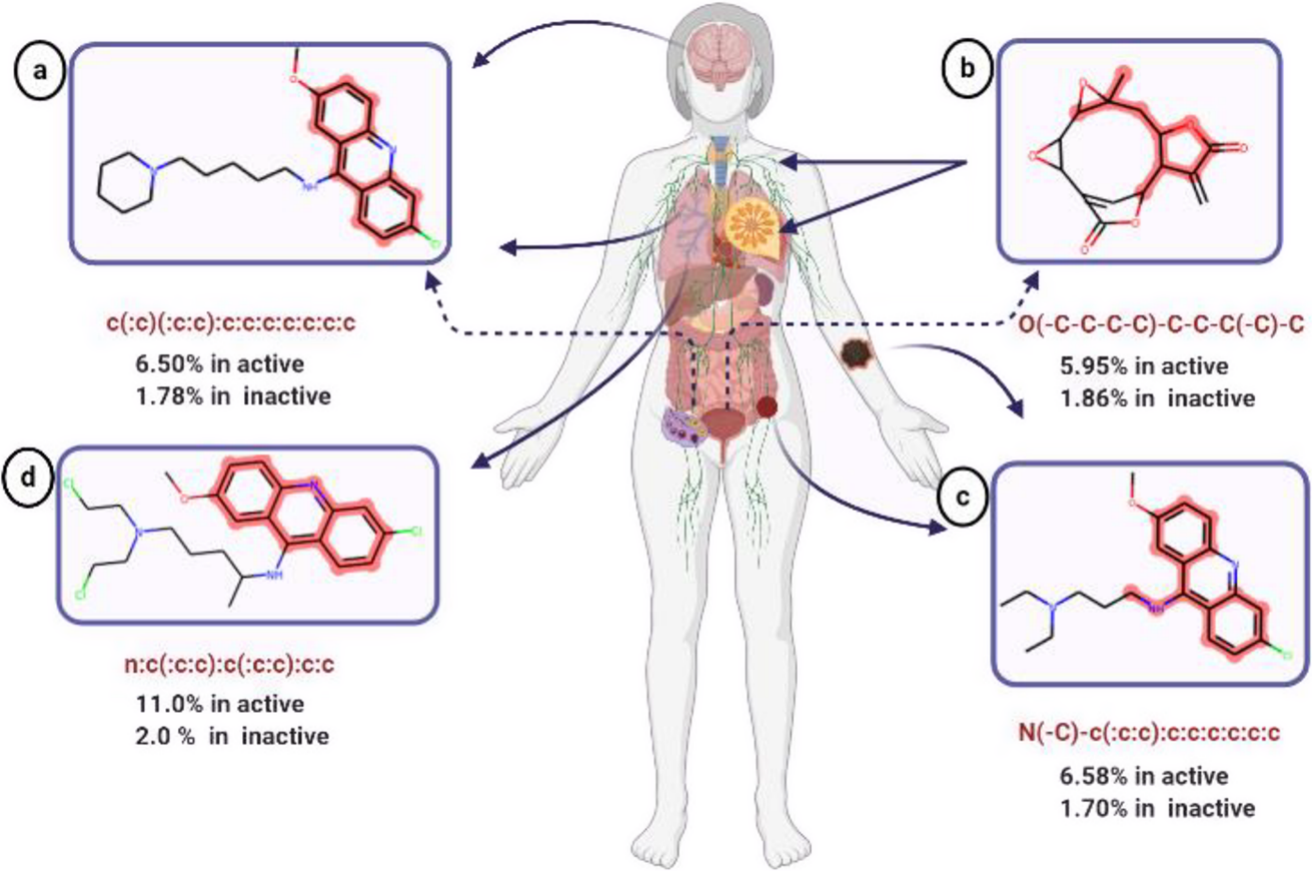
Molecular substructure enrichment in compounds with anticancer activity. (A) The first fragment occurred 6.50% in active and 1.78% in inactive molecules, tested against the CNS, ovarian, and non-small-cell lung cancer cell panels (lines). (B) The second fragment occurred 5.95% in active and 1.86% in inactive molecules, tested against leukemia, renal, and breast cancer cell panels. (C) The third fragment was identified in 6.58% in active and 1.70% in inactive molecules, tested against prostate (not shown), melanoma, and colon cancer cell lines. (D) The fourth fragment was found in 11% in active and 2.0% in inactive compounds, tested against small lung cancer cell lines.

Four common substructures were found to be occurring more frequently in active molecules in comparison with inactive molecules. The active molecules were found to be enriched in benzene rings. The analysis presented in Figure 2 depicts the commonly occurring substructures.

The first substructure (Figure 2A) is naphthalene, which is a simple polycyclic aromatic hydrocarbon. Chemical compounds derived from naphthalene are known to display different biological activities, which include anticancer, antibacterial, and anti-inflammatory activities.^26^ Additionally, several studies have reported that naphthalene derivatives could induce cell apoptosis in many types of cancer cells, such as colon cancer cells, breast cancer cells, melanoma cells, and lung cancer cells.^26−28^

The second substructure (Figure 2B) is the lactone structure. Several compounds containing lactone exhibited remarkable *in vitro* cytotoxic activity as well as apoptosis against non-small cell lung, breast, and leukemia cancer cell lines.^29,30^

The third substructure (Figure 2C) is the *N*-methyl aniline structure, which is used for designing Src kinase inhibitors. Small molecules containing *N*-methyl aniline have exhibited potential anticancer activity against different tumor types.^31^

The fourth substructure (Figure 2D) is quinoline, which is an aromatic heterocyclic compound. It consists of a benzene and a pyridine ring. Also, it is a part of the known topoisomerase II inhibitor voreloxin.^32^ Derivatives of quinoline have shown significant anticancer activity by different mechanisms including apoptosis and growth inhibition through cell cycle arrest.^33^

### Predicting Small Molecules with Anticancer Activity

To predict anticancer pharmacodynamic properties of small molecules, as well as their bioactivity, different supervised machine learning algorithms were applied to train a classification predictive model, using evidence from the distance-based graph signatures and more general physicochemical properties. A diverse data set of 18,369 experimentally characterized molecules with anticancer properties screened against NCI-60 cancer cell lines were employed, including 8565 active and 9804 inactive molecules.

In the NCI-60 DTP project, molecule screening was carried out in two stages to discover potential anticancer activity. Initially, all compounds were screened against 60 cancer cell lines at 5–10 molar concentrations. Furthermore, compounds exhibiting significant growth inhibition were evaluated on the NCI-60 cell panel at five different concentration levels.

**Figure 3:**
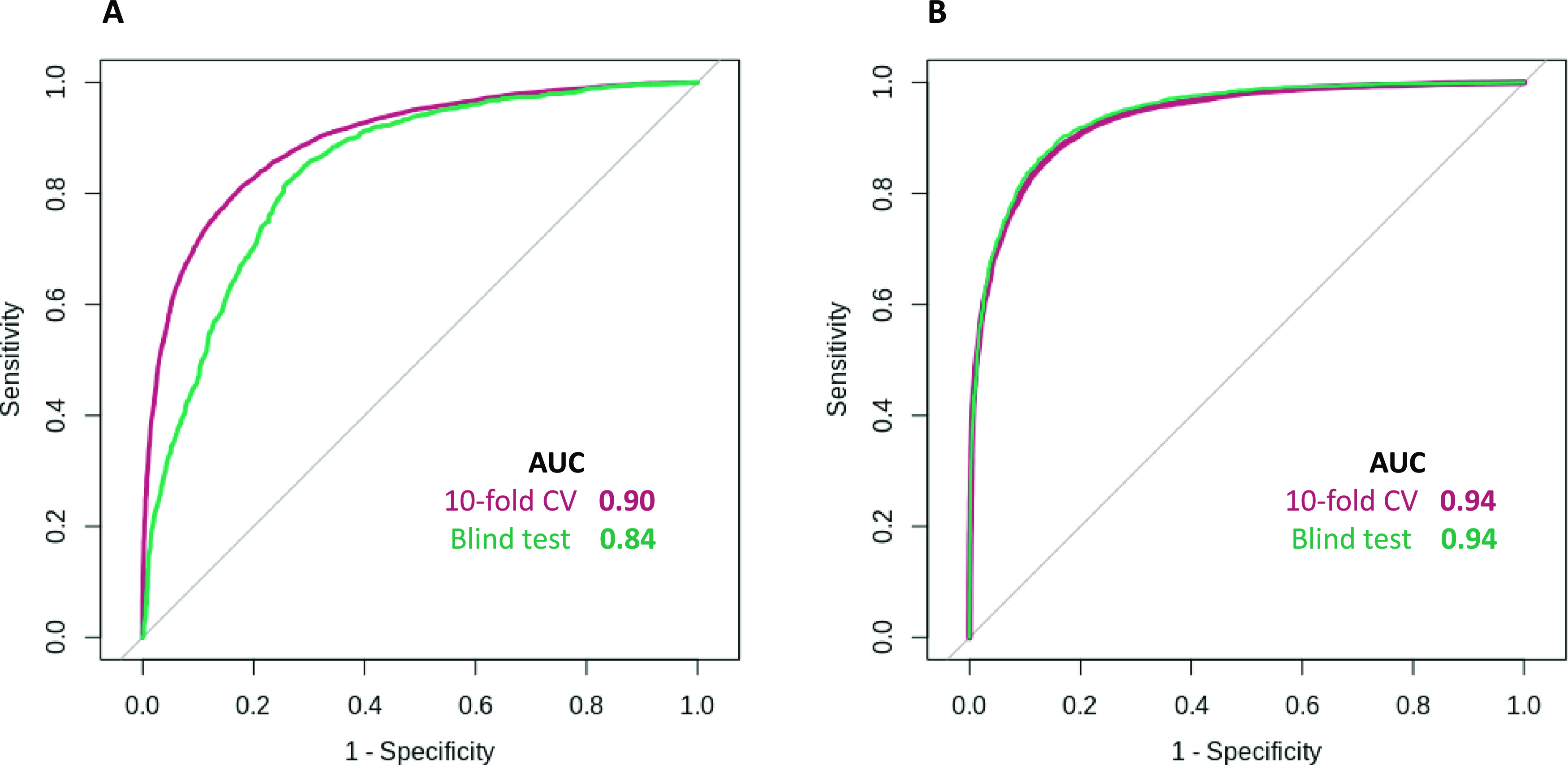
Receiver operating characteristic (ROC) curves of the pdCSM-cancer classification model. Our predictive model accurately identified active molecules with AUC > 0.84 on cross-validation and blind tests. (A) Performance of pdCSM-cancer on the CDRUG data. (B) Performance of pdCSM-cancer predictor on the updated NCI-60 data.

After feature selection, our final model achieved an accuracy of 0.86 on 10-fold cross-validation, with an area under the ROC curve (AUC) of 0.90 and a precision of 0.85 (Figure 3A), significantly outperforming the alternative approach CDRUG developed using the same data (Table 1). This was comparable to the performance across the non-redundant blind test, achieving an accuracy of 0.77, AUC of 0.84, and precision of 0.79, providing confidence in the generalizability of the final model (Figure 3).

**Table 1:** Comparative Performance on Cross-Validation between the pdCSM-cancer Classification Model and Another Available Approach, CDRUG^a^

method	MCC	sensitivity	FPR	accuracy	AUC
pdCSM-cancer (updated NCI-60 data)	0.72	0.84	0.13	0.86	0.94
pdCSM-cancer (CDRUG data)	0.70	0.85	0.15	0.86	0.90
CDRUG^10^	*	0.81	0.20	*	0.87

a Asterisk: MCC and accuracy predictive scores were not reported by CDRUG, neither the prediction matrix (i.e., predicted and actual values for each molecule).

Furthermore, we tested the performance of our final model on the updated NCI-60 data, where it achieved an accuracy of 0.86 on 10-fold cross-validation, AUC of 0.94, and precision of 0.84 (Figure 3B). On the non-redundant blind test, it achieved an accuracy of 0.87, AUC of 0.94, and precision of 0.85. The model performance was consistent on both data sets (the updated NCI-60 and CDRUG data), which shows the robustness of the method (Table 1).

A two-sample Kolmogorov–Smirnov test was conducted on all the features used in the final model to determine which distinct features of the compounds translate into anticancer activity. We found that molecules with anticancer activity tended to have a high frequency of lactone and pyridine rings (*p*-value < 1.9 e-07). Interestingly, the inactive compounds tended to have a higher frequency of amides. These characteristics were further validated by substructure mining. Figure S2 depicts the top discriminative features of active compounds compared to inactive compounds.

### Cell Line-Specific Activity Prediction

While the performance of our approach to identify likely generic anticancer compounds provided further confidence into the potential of graph-based signatures to provide biological insight, cancer is not a single disease. We therefore applied this approach to predict the anticancer activity (GI50%) against each of the 74 specific cancer cell lines. The final models achieved Pearson’s correlation coefficients ranging from 0.58 (for SNB-78 cell line) to 0.74 (for P388 cell line) across 10-fold cross validation (Table 2 and Table S2 and Figures S3–S12). Similarly, Cortés-Ciriano and colleagues modeled the 50% growth inhibition (GI50%) of thousands of compounds screened against 59 cancer cell lines of the NCI60 panel, and by combining chemical and biological information, their models achieved a mean Pearson’s correlations ranging from 0.41 (for LOCCO: leave-one-compound-out model) and 0.88 (for LOTO: leave-one-tissue-out model).^34^ The results obtained for our final models are comparable to the performance reported previously.

**Table 2:** Performance of the Final pdCSM-Cancer Regression Models on Cross-Validation and Blind Test Sets

tissue	cell lines	Pearson (CV)	Pearson (blind test)
CNS	SF-268	0.66	0.59
	SF-295	0.68	0.59
	SF-539	0.66	0.59
	SNB-19	0.66	0.61
	SNB-75	0.63	0.59
	SNB-78	0.61	0.52
	U251	0.69	0.63
	XF-498	0.60	0.51
breast	BT-549	0.65	0.56
	HS-578 T	0.64	0.54
	MCF7	0.69	0.59
	MDA-MB-231_ATCC	0.68	0.58
	MDA-MB-468	0.58	0.49
	T-47D	0.64	0.56
colon	COLO-205	0.67	0.58
	DLD-1	0.66	0.59
	HCC-2998	0.63	0.63
	HCT-116	0.69	0.61
	HCT-15	0.63	0.56
	HT29	0.67	0.65
	KM12	0.66	0.59
	KM20L2	0.62	0.53
	SW-620	0.69	0.59
leukemia	CCRF-CEM	0.65	0.59
	HL-60 TB	0.63	0.56
	K-562	0.66	0.57
	MOLT-4	0.67	0.59
	P388_ADR	0.69	0.67
	P388	0.74	0.65
	RPMI-8226	0.63	0.59
	SR	0.63	0.62
ovarian	IGROV1	0.66	0.57
	NCI_ADR-RES	0.65	0.56
	OVCAR-3	0.67	0.58
	OVCAR-4	0.63	0.62
	OVCAR-5	0.64	0.54
	OVCAR-8	0.68	0.59
	SK-OV-3	0.65	0.57
prostate	DU-145	0.67	0.58
	PC-3	0.69	0.59
renal	786-0	0.65	0.56
	A498	0.64	0.63
	ACHN	0.68	0.59
	CAKI-1	0.65	0.56
	RXF-393	0.65	0.56
	RXF-631	0.66	0.63
	SN12C	0.65	0.59
	SN12K1	0.74	0.65
	TK-10	0.64	0.61
	UO-31	0.65	0.55
non-small cell lung	A549_ATCC	0.68	0.58
	EKVX	0.62	0.53
	HOP-18	0.54	0.48
	HOP-62	0.65	0.59
	HOP-92	0.60	0.57
	LXFL-529	0.67	0.59
	NCI-H226	0.64	0.54
	NCI-H23	0.69	0.59
	NCI-H322M	0.64	0.67
	NCI-H460	0.69	0.63
	NCI-H522	0.67	0.57
small cell lung	DMS-114	0.67	0.58
	DMS-273	0.58	0.48
melanoma	LOX-IMVI	0.67	0.59
	M14	0.66	0.58
	M19-MEL	0.66	0.59
	MALME-3 M	0.64	0.59
	MDA-MB-435	0.68	0.59
	MDA-N	0.66	0.61
	SK-MEL-28	0.66	0.59
	SK-MEL-2	0.65	0.56
	SK-MEL-5	0.66	0.64
	UACC-257	0.65	0.59
	UACC-62	0.67	0.59

**Figure 4:**
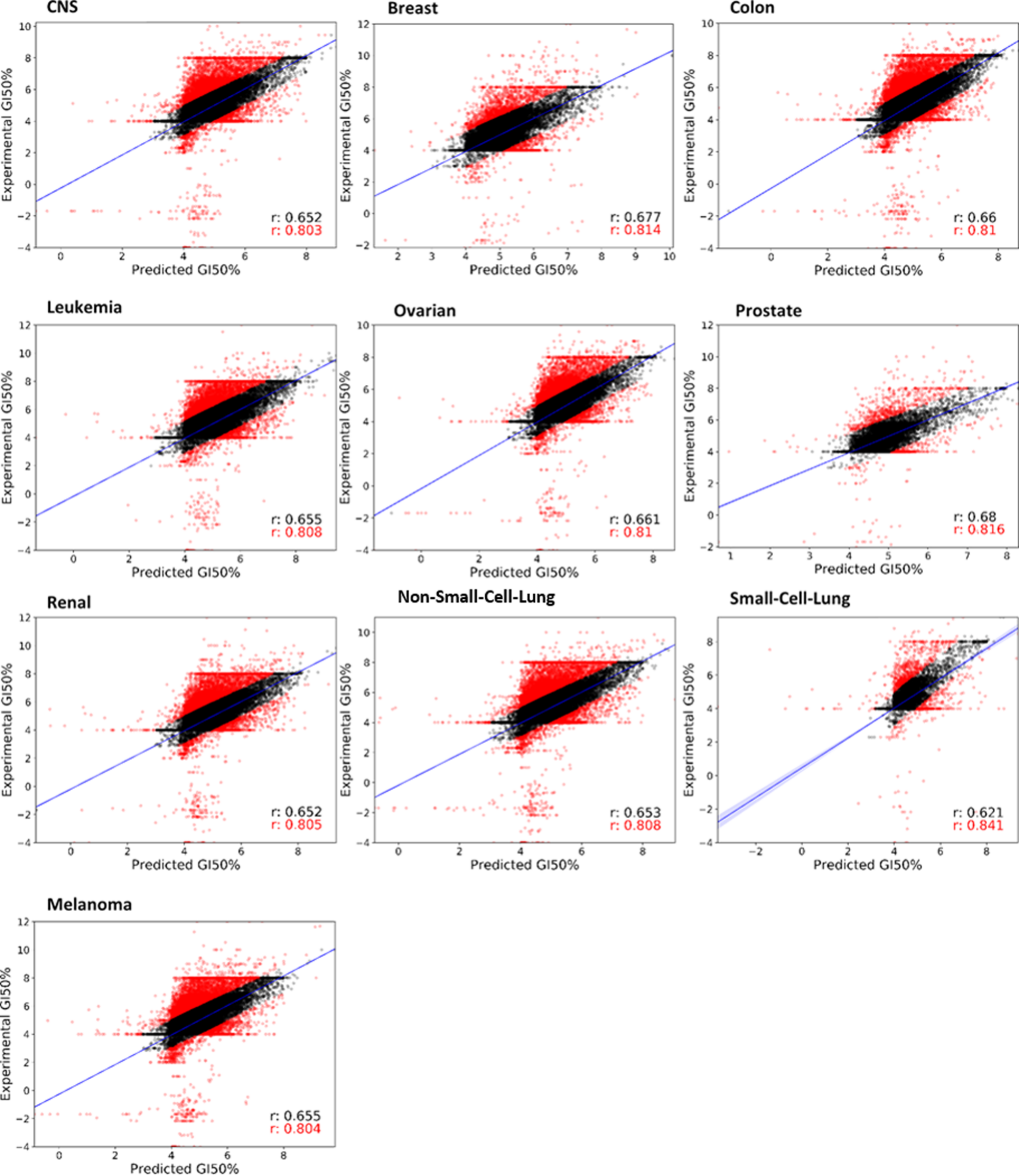
pdCSM-cancer regression performance on cross-validation. Scatter plots between experimental and predicted GI50% values, given in −log_10_(molar), for each of the nine cancer cell line (panels) models are displayed. Pearson’s correlation coefficient (*r*) is shown for each scatter plot (in black for 90% of the data, after 10% of outliers (depicted in red) were removed).

Figure 4 depicts the performance in terms of predicted *versus* experimental GI50% values^35^ for each model for 10-fold cross-validation, also highlighting performance on 90% of the data (i.e*.*, after 10% outlier removal).

To further validate the models, these were evaluated on independent blind tests sets. The performance was observed to be consistent between cross-validation and blind tests, indicating the generalization capabilities of the models. For blind tests, the models achieved Pearson’s correlations ranging from 0.48 (DMS-273 cell line) and 0.67 (P388_ADR cell line) (Table 2 and Figures S13–S22).

Comparison of the distributions of the predicted *versus* experimental GI50% values^35^ for each model of the 74 specific cancer cell lines revealed that there was a significant overlap of compounds with an experimental −log_10_(GI50%) value of 4 (Figures S3–S22), indicating a low anti-proliferative activity against all types of cancer cell lines tested, which might impose a bias and, as a consequence, a challenge for the regression models in predicting anticancer activity. To account for this, a general classifier model was built that is capable of predicting anticancer and non-anticancer molecules, as described above. To the best of our knowledge, this is the first effort of developing cancer cell line-specific bioactivity predictors using the NCI-60 panels.

Compounds that are active on a given tissue type could display similar molecular signatures against other cancer types and their activity could be a reflection of similar chemical structures.^7^ Therefore, we examined whether cancer types could be clustered based on common molecular features between compounds. We performed a clustering analysis based on *k*-means and hierarchical agglomerative clustering on the 74 specific cancer cell lines. Interestingly, the clustering analysis revealed that the cell lines per tissue type do not cluster together, apart from a group of melanoma cell lines that tended to form a small cluster. Nevertheless, even if the cell line has a distinct identity, the properties of a given data set might be subject to sampling biases or may not match the cancer type of origin. The latter may be because tumors that are supposed to arise in a particular tissue could be a metastatic lesion arising from a distal site. Consequently, cell lines originating from such tumors might have a different tissue type identification from that specified at isolation, creating a cause of mislabeling.^36^Figure S23 shows a heatmap of the distinctive molecular features between different cancer cell lines.

### pdCSM-cancer Web Server

To provide the scientific community with pdCSM-cancer functionalities, we have implemented an easy-to-use web interface freely available at http://biosig.unimelb.edu.au/pdcsm_cancer. Users can predict the anticancer activity and GI50% values of their molecules of interest by submitting either a single molecule or multiple molecules as a batch file by presenting molecules as SMILES strings.

Additionally, it allows users to calculate the pharmacokinetic properties of their chosen molecules using the pkCSM tool (Figure S24).

## Conclusions

Here, we have presented pdCSM-cancer, an optimized graph-based signature approach for predicting safe, efficient, and biologically active compounds against multiple cancer types. pdCSM-cancer is capable of quantitatively predicting small molecules that are likely to be active against one or multiple cancer cell lines.

Using graph-based signatures, we have built and implemented 74 regression models capable of quantitatively assessing molecule bioactivity and a predictive classification model with categorical outputs for predicting anticancer activity for novel molecules as well as their physicochemical properties. We have made pdCSM-cancer available through a user-friendly web server at http://biosig.unimelb.edu.au/pdcsm_cancer.

We believe that pdCSM-cancer would be a valuable tool for augmenting screening approaches to identifying novel anticancer drugs, increasing hit rates and reducing costs. This will hopefully facilitate the process of drug discovery by enabling the rapid design and optimization of compounds with anticancer properties.

As future work, we intend to investigate the association of combined drugs^37,38^ (i.e., molecules) to improve the efficiency of cancer treatments. Our further study would seek to understand how the anticancer bioactivity of a certain compound changes when it interacts with others. In addition, to incorporate more interpretability into pdCSM-cancer, we aim to predict not only the anticancer property for that particular drug but also hierarchically its respective target(s).^39−42^ With this, drug treatments may be more understandable in terms of efficiency and side effects, for instance. Finally, further development of a molecular generative model (e.g., conditional variational autoencoder)^43^ will be carried out. Autoencoders would provide the ability to test/screen drugs that are out of the scope of the database (e.g., NCI-60). This would likely yield novel drugs with anticancer properties, which have not been tested yet.

## Methods

### Data Sets

All datasets were curated from the NCI-60 Development Therapeutics Project (DTP),^12^ which has thousands of small molecules analyzed against NCI-60 human cancer cell lines. In this fashion, to develop a general bioactivity predictor, first, a dataset of bioactive molecules with anticancer properties were constructed from an updated version of NCI-60 data (June 2020). The data for constructing the updated version of the anticancer bioactivity dataset can be retrieved from NCI DTP on NCI-60 Growth Inhibition Data.^23^ The construction of this first dataset was based on the same methodology used by Li and Huang’s study^10^ and comprehends 12,646 active and 15,777 inactive molecules after merging with Li and Huang’s previous benchmark data. Second, for direct comparison with the CDRUG approach, Li and Huang’s dataset was solely employed to test the predictive performance of pdCSM-cancer. This benchmark data comprises 8565 active and 9804 inactive molecules.

It is worth noting that the bioactivity in both datasets is defined based on one dose and dose-response data. On these dose data, a molecule is considered as inactive if the average of the growth inhibition rate is lower than 5% at a dose of 10^–5^ mol on one dose data. On the other hand, a molecule is set as active if the average of the growth inhibition rate is higher than 50% at a dose of 10^–5^ mol on dose-response data.

Experimental GI50% (50% of cell growth inhibition) values, given in −log_10_(molar), for the NCI-60 human cancer cell lines were also collected from the NCI-60 Development Therapeutics Project,^38^ including 15,636 experimental results against the 60 different cancer cell lines (plus 14 additional cancer cell lines) derived from nine tumor types. This data was used as evidence to train cell line-specific learning models for regression tasks and assess their final predictive/generalization performance. The negative logarithm of the GI50% values of each molecule was combined (based on NCI identifiers) for each cancer cell line to generate tissue-specific data sets, which included at least 7677 unique molecules.

The bioactivity and GI50% datasets were divided into a non-redundant training (80%) and blind test (20%) to train and evaluate the predictive/generalization performance of the predictive models used for the classification and regression tasks. Molecules were clustered on Morgan/Circular fingerprints with the Butina algorithm using Tanimoto similarity (at a 0.6 similarity level) using RDKit^46^ to make sure that similar molecules were either on training or test sets. All datasets used in this study are available at http://biosig.unimelb.edu.au/pdcsm_cancer/data.

### Graph-Based Signatures to Represent Small Molecules

Graph modeling is a well-established mathematical representation used to model chemical entities, which relies on the structural fingerprints of molecular descriptors to determine the relationships between molecular structures and their biological activities. These signatures have been proven to be a general and powerful tool to model the physicochemical properties of small molecules^14^ and other biological entities.^16,44,45^ We have previously proposed the concept of graph-based signatures to represent protein structure geometry and the molecular interactions with their binding partners as graphs.^19,46−49^ These were successfully used and adapted to train and test many different machine learning models, such as the prediction and optimization of pharmacokinetic and toxicity properties using a pkCSM tool.^15^ We employed and adapted these distance-based signatures to model small-molecule chemistry, enabling the prediction of their anticancer properties.

There are two key components of the graph-based signatures: (i) compound physicochemical properties obtained via the RDKit cheminformatics library^50^ and (ii) distance-based signatures, described as a cumulative distribution function of distances in atoms defined based on their corresponding physicochemical properties (pharmacophores) (Table S1). The distance-based patterns are encoded in a small-molecule graph-based signature that was adjusted from the Cutoff Scanning matrix method.^51^ In this approach, each dimension of the molecular signature expresses the number of atoms (characterized by pharmacophore class) within a particular distance in the graph. The cost of the shortest path is based on the shortest distance between any two nodes in the molecular graph, calculated by Johnson’s algorithm. It is described as the total weights of the edges on the path, where all the edges are considered to have unitary weight (Figure S25). Hence, the value of the shortest path is expressed as the number of edges in it.^15^ Using the graph-based signature approach, a total of 264 features were obtained and used to train and test the predictive models.

It is worth noting that there are other ways to represent small molecules in order to build machine learning for molecular prediction. For instance, one of the current approaches is based on deep feature generation through graph neural networks (GNN).^52^ Although being a successful approach, GNN-generated features have, as a major drawback, the lack of an inherent interpretability, which is a natural aspect from the graph-based signatures. Accordingly, graph-based signature features were first preferred for pdCSM-cancer than others. In future work, other types of features (e.g., deep GNN features) will be incorporated into the pdCSM-cancer models after carrying out an analysis of their predictive benefits.

### Feature Selection

Selecting the best set of features to train predictive models is known to be a challenging problem. A bottom-up greedy feature selection method was employed to reduce the redundancy, noise, and low representativity of the 264 graph-based signature features obtained to represent molecules.

This method starts with zero features, by considering each feature independently, adds them one by one in accordance to a machine learning model, and keeps only the set of features with the most prominent performance metric (e.g., Pearson’s correlation coefficient) on that particular model.

### Model Selection and Evaluation

To obtain a predictive classification model, we first evaluated several learning algorithms, including random forest, extremely randomized trees, gradient boosting, *k*-nearest neighbors, and extreme gradient boosting, using the implementation available on the Scikit-learn library.^53^ Random forest was the one with the best predictive performance on 10-fold cross-validation over the training set after greedy feature selection. Therefore, the random forest classifier was used as our final model.

Random forest is a powerful ensemble-learning algorithm that generates multiple models of decision trees from a randomly chosen subset of the training set. It then aggregates the votes from various decision trees to determine the most voted class of the test object.^54^ The predictive model was trained and assessed using 10-fold cross-validation and a non-redundant blind test. The model performance was evaluated using different evaluation metrics, which include accuracy, precision, and the area under the ROC curve (AUC). AUC is an effective measure to evaluate a model’s performance in a classification task at various threshold settings. It is based on the ROC curve, which is plotted with the true positive rate (TPR) against the false positive rate (FPR). Higher AUC means that the model is robust and capable of discriminating between the two classes: active and inactive. AUC uses values between 0 and 1. Therefore, an accurate model would have an AUC of 1, and an AUC of 0.5 indicates that the model is a random classifier.

In the regression counterpart of this work, we also analyzed different regression supervised learning methods to build 74 models for predicting the GI50% values, including gradient boosting, extreme gradient boosting, random forest, extremely randomized trees, and adaptive boosting, which were applied via the Scikit-learn library.^55^ Pearson’s correlation coefficient, RMSE, and Kendall’s correlation coefficient were employed to select the model with the best performance (Table S2) after greedy feature selection. A 10-fold cross-validation procedure and non-redundant blind test were employed to evaluate the performance of the predictive models. To examine the effect of potential outliers, the model’s performance was evaluated on 100% and also on 90% of the data, which can be interpreted as the full data set minus the 10% worst predicted data points (i.e*.*, the points that fall away from the regression line). For the datasets, the ensemble methods, extremely randomized trees and random forest, were found to be the best performing algorithms (Table S2).

### Substructure Mining

Molecular Substructure miner (MoSS)^25^ was used to investigate the substructure enrichment in molecules with anticancer activity. We examined frequent substructures in a focused group including active molecules, in comparison to a complementary set (inactive molecules). To find the frequency of substructure enrichment in a set of molecules, we used a minimum support (frequency) of 10% (default) in the focus set and a 2% of the maximum support (frequency) in complement. Also, the threshold for the split was specified as 0.5.

### Web Server Development

The web server front end was developed via Bootstrap framework version 3.3.7, and the back end was built in Python 2.7 with the use of the Flask framework (version 0.12.3). It is hosted on a Linux server running Apache.

## Supporting Information Available

Physiochemical properties and distribution of compounds; pdCSM-cancer blind test and cross-validation results; heatmap of common molecular properties in the NCI60 panels; pdCSM-cancer web server interface; complementary features applied in pdCSM-cancer development; performance of pdCSM-cancer using different evaluation metrics; and graph-based signatures calculations (PDF)

## Notes

All data sets used in this study to train and validate the predictive models are freely available at http://biosig.unimelb.edu.au/pdcsm_cancer/data. Our predictive model pdCSM-cancer is freely available as a user-friendly web interface at http://biosig.unimelb.edu.au/pdcsm_cancer.

## References

[ref1] World Health O. Cancer; Https://Www.Who.Int/En/News-Room/Fact-Sheets/Detail/Cancer. *WHO/CDS/CSR/GAR/2003.11* 2003.

[ref2] McQuade R. M.; Stojanovska V.; Bornstein J. C.; Nurgali K. Colorectal Cancer Chemotherapy: The Evolution of Treatment and New Approaches. Curr. Med. Chem. 2017, 1537 10.2174/0929867324666170111152436. 28079003

[ref3] Quinn D. I.; Sandler H. M.; Horvath L. G.; Goldkorn A.; Eastham J. A. The Evolution of Chemotherapy for the Treatment of Prostate Cancer. Annl Oncol. 2017, 2658 10.1093/annonc/mdx348. 29045523

[ref4] Kibria G.; Hatakeyama H.; Harashima H. Cancer Multidrug Resistance: Mechanisms Involved and Strategies for Circumvention Using a Drug Delivery System. Arch. Pharmacal Res. 2014, 4 10.1007/s12272-013-0276-2. 24272889

[ref5] Singh H.; Kumar R.; Singh S.; Chaudhary K.; Gautam A.; Raghava G. P. S. Prediction of Anticancer Molecules Using Hybrid Model Developed on Molecules Screened against Nci-60 Cancer Cell Lines. BMC Cancer 2016, 16, 77 10.1186/s12885-016-2082-y. 26860193 PMC4748564

[ref6] Cui W.; Aouidate A.; Wang S.; Yu Q.; Li Y.; Yuan S. Discovering Anti-Cancer Drugs Via Computational Methods. Front. Pharmacol. 2020, 11, 733 10.3389/fphar.2020.00733. 32508653 PMC7251168

[ref7] Reymond J. L.; Awale M. Exploring Chemical Space for Drug Discovery Using the Chemical Universe Database. ACS Chem. Neurosci. 2012, 649 10.1021/cn3000422. 23019491 PMC3447393

[ref8] Moffat J. G.; Rudolph J.; Bailey D. Phenotypic Screening in Cancer Drug Discovery-Past, Present and Future. Nat. Rev. Drug Discovery 2014, 588 10.1038/nrd4366. 25033736

[ref9] Hoelder S.; Clarke P. A.; Workman P. Discovery of Small Molecule Cancer Drugs: Successes, Challenges and Opportunities. Mol. Oncol. 2012, 155 10.1016/j.molonc.2012.02.004. 22440008 PMC3476506

[ref10] Li G. H.; Huang J. F. Cdrug: A Web Server for Predicting Anticancer Activity of Chemical Compounds. Bioinformatics 2012, 28, 3334–3335. 10.1093/bioinformatics/bts625. 23080119

[ref11] Menden M. P.; Iorio F.; Garnett M.; McDermott U.; Benes C. H.; Ballester P. J.; Saez-Rodriguez J. Machine Learning Prediction of Cancer Cell Sensitivity to Drugs Based on Genomic and Chemical Properties. PLoS One 2013, 8, e61318 10.1371/journal.pone.0061318. 23646105 PMC3640019

[ref12] Shoemaker R. H. The Nci60 Human Tumour Cell Line Anticancer Drug Screen. Nat. Rev. Cancer 2006, 6, 813–823. 10.1038/nrc1951. 16990858

[ref13] Kumar R.; Chaudhary K.; Singla D.; Gautam A.; Raghava G. P. S. Designing of Promiscuous Inhibitors against Pancreatic Cancer Cell Lines. Sci. Rep. 2015, 4, 4668 10.1038/srep04668. PMC3985076 24728108

[ref14] Pires D. E. V.; Ascher D. B. Csm-Lig: A Web Server for Assessing and Comparing Protein-Small Molecule Affinities. Nucleic Acids Res. 2016, 44, W557–W561. 10.1093/nar/gkw390. 27151202 PMC4987933

[ref15] Pires D. E. V.; Blundell T. L.; Ascher D. B. Pkcsm: Predicting Small-Molecule Pharmacokinetic and Toxicity Properties Using Graph-Based Signatures. J. Med. Chem. 2015, 58, 4066–4072. 10.1021/acs.jmedchem.5b00104. 25860834 PMC4434528

[ref16] Pires D. E. V.; Ascher D. B. Mycocsm: Using Graph-Based Signatures to Identify Safe Potent Hits against Mycobacteria. J. Chem. Inf. Model. 2020, 3450 10.1021/acs.jcim.0c00362. 32615035

[ref17] Pires D. E. V.; Stubbs K. A.; Mylne J. S.; Ascher D. B. Designing Safe and Potent Herbicides with the Cropcsm Online Resource. bioRxiv 2020, 2020.11.01.364240.

[ref18] Kaminskas L. M.; Pires D. E. V.; Ascher D. B. Dendpoint: A Web Resource for Dendrimer Pharmacokinetics Investigation and Prediction. Sci. Rep. 2019, 9, 15465 10.1038/s41598-019-51789-3. 31664080 PMC6820739

[ref19] Rodrigues C. H. M.; Myung Y.; Pires D. E. V.; Ascher D. B. Mcsm-Ppi2: Predicting the Effects of Mutations on Protein-Protein Interactions. Nucleic Acids Res. 2019, W338 10.1093/nar/gkz383. 31114883 PMC6602427

[ref20] Jiang J.; Wang R.; Wei G. W. Ggl-Tox: Geometric Graph Learning for Toxicity Prediction. J. Chem. Inf. Model. 2021. 10.1021/acs.jcim.0c01294 PMC8155789 33719422

[ref21] Wu Z.; Ramsundar B.; Feinberg E. N.; Gomes J.; Geniesse C.; Pappu A. S.; Leswing K.; Pande V. Moleculenet: A Benchmark for Molecular Machine Learning. Chem. Sci. 2018, 9, 513–530. 10.1039/C7SC02664A. 29629118 PMC5868307

[ref22] Winter R.; Montanari F.; Noe F.; Clevert D. A. Learning Continuous and Data-Driven Molecular Descriptors by Translating Equivalent Chemical Representations. Chem. Sci. 2019, 10, 1692–1701. 10.1039/C8SC04175J. 30842833 PMC6368215

[ref23] https://wiki.nci.nih.gov/display/NCIDTPdata/NCI-60+Data+Download+-+Previous+Releases

[ref24] Lipinski C. A.; Lombardo F.; Dominy B. W.; Feeney P. J. Experimental and Computational Approaches to Estimate Solubility and Permeability in Drug Discovery and Development Settings. Adv. Drug Delivery Rev. 2001, 3 10.1016/S0169-409X(00)00129-0. 11259830

[ref25] Borgelt C.; Meinl T.; Berthold M. Moss: A Program for Molecular Substructure Mining. Proceedings of the ACM SIGKDD International Conference on Knowledge Discovery and Data Mining*;* ACM Digital Library 2005, 6–15.

[ref26] Xu W. T.; Shen G. N.; Luo Y. H.; Piao X. J.; Wang J. R.; Wang H.; Zhang Y.; Li J. Q.; Feng Y. C.; Zhang Y.; Zhang T.; Wang S. N.; Wang C. Y.; Jin C. H. New Naphthalene Derivatives Induce Human Lung Cancer A549 Cell Apoptosis Via Ros-Mediated Mapks, Akt, and Stat3 Signaling Pathways. Chem.-Biol. Interact. 2019, 148 10.1016/j.cbi.2019.03.004. 30871965

[ref27] Kretschmer N.; Rinner B.; Deutsch A. J. A.; Lohberger B.; Knausz H.; Kunert O.; Blunder M.; Boechzelt H.; Schaider H.; Bauer R. Naphthoquinones from Onosma Paniculata Induce Cell-Cycle Arrest and Apoptosis in Melanoma Cells. J. Nat. Prod. 2012, 75, 865–869. 10.1021/np2006499. 22530779 PMC3361261

[ref28] Hae J. K.; Jung Y. M.; Young J. C.; Kyung H. C.; Sung W. H.; Mie Y. K. Effects of a Naphthoquinone Analog on Tumor Growth and Apoptosis Induction. Arch. Pharmacal Res. 2003, 26, 405–410. 10.1007/BF02976698 12785737

[ref29] Ma X.; Wu K.; Xu A.; Jiao P.; Li H.; Xing L. The Sesquiterpene Lactone Eupatolide Induces Apoptosis in Non-Small Cell Lung Cancer Cells by Suppressing Stat3 Signaling. Environ. Toxicol. Pharmacol. 2021, 81, 103513 10.1016/j.etap.2020.103513. 33091599

[ref30] Gach-Janczak K.; Drogosz-Stachowicz J.; Dlugosz-Pokorska A.; Jakubowski R.; Janecki T.; Szymanski J.; Janecka A. A New Hybrid Delta-Lactone Induces Apoptosis and Potentiates Anticancer Activity of Taxol in Hl-60 Human Leukemia Cells. Molecules 2020, 25, 1479 10.3390/molecules25071479. PMC7180485 32218198

[ref31] Rao V. K.; Chhikara B. S.; Shirazi A. N.; Tiwari R.; Parang K.; Kumar A. 3-Substitued Indoles: One-Pot Synthesis and Evaluation of Anticancer and Src Kinase Inhibitory Activities. Bioorg. Med. Chem. Lett. 2011, 21, 3511–3514. 10.1016/j.bmcl.2011.05.010. 21612925

[ref32] Hawtin R. E.; Stockett D. E.; Byl J. A. W.; McDowell R. S.; Nguyen T.; Arkin M. R.; Conroy A.; Yang W.; Osheroff N.; Fox J. A. Voreloxin Is an Anticancer Quinolone Derivative That Intercalates DNA and Poisons Topoisomerase Ii. PLoS One 2010, 5, e10186 10.1371/journal.pone.0010186. 20419121 PMC2855444

[ref33] Katariya K. D.; Shah S. R.; Reddy D. Anticancer, Antimicrobial Activities of Quinoline Based Hydrazone Analogues: Synthesis, Characterization and Molecular Docking. Bioorg. Chem. 2020, 103406 10.1016/j.bioorg.2019.103406. 31718889

[ref34] Cortes-Ciriano I.; van Westen G. J.; Bouvier G.; Nilges M.; Overington J. P.; Bender A.; Malliavin T. E. Improved Large-Scale Prediction of Growth Inhibition Patterns Using the Nci60 Cancer Cell Line Panel. Bioinformatics 2015, 32, 85–95. 10.1093/bioinformatics/btv529. 26351271 PMC4681992

[ref35] Holbeck S. L.; Collins J. M.; Doroshow J. H. Analysis of Food and Drug Administration-Approved Anticancer Agents in the Nci60 Panel of Human Tumor Cell Lines. Mol. Cancer Ther. 2010, 9, 1451–1460. 10.1158/1535-7163.MCT-10-0106. 20442306 PMC2868078

[ref36] Salvadores M.; Fuster-Tormo F.; Supek F. Matching Cell Lines with Cancer Type and Subtype of Origin Via Mutational, Epigenomic, and Transcriptomic Patterns. Sci. Adv. 2020, 6, eaba1862 10.1126/sciadv.aba1862. 32937430 PMC7458440

[ref37] Zagidullin B.; Aldahdooh J.; Zheng S.; Wang W.; Wang Y.; Saad J.; Malyutina A.; Jafari M.; Tanoli Z.; Pessia A.; Tang J. Drugcomb: An Integrative Cancer Drug Combination Data Portal. Nucleic Acids Res. 2019, 47, W43–W51. 10.1093/nar/gkz337. 31066443 PMC6602441

[ref38] Holbeck S. L.; Camalier R.; Crowell J. A.; Govindharajulu J. P.; Hollingshead M.; Anderson L. W.; Polley E.; Rubinstein L.; Srivastava A.; Wilsker D.; Collins J. M.; Doroshow J. H. The National Cancer Institute Almanac: A Comprehensive Screening Resource for the Detection of Anticancer Drug Pairs with Enhanced Therapeutic Activity. Cancer Res. 2017, 77, 3564–3576. 10.1158/0008-5472.CAN-17-0489. 28446463 PMC5499996

[ref39] Gfeller D.; Grosdidier A.; Wirth M.; Daina A.; Michielin O.; Zoete V. Swisstargetprediction: A Web Server for Target Prediction of Bioactive Small Molecules. Nucleic Acids Res. 2014, 42, W32–W38. 10.1093/nar/gku293. 24792161 PMC4086140

[ref40] Daina A.; Michielin O.; Zoete V. Swisstargetprediction: Updated Data and New Features for Efficient Prediction of Protein Targets of Small Molecules. Nucleic Acids Res. 2019, 47, W357–W364. 10.1093/nar/gkz382. 31106366 PMC6602486

[ref41] Hamad S.; Adornetto G.; Naveja J. J.; Chavan Ravindranath A.; Raffler J.; Campillos M. Hitpickv2: A Web Server to Predict Targets of Chemical Compounds. Bioinformatics 2019, 35, 1239–1240. 10.1093/bioinformatics/bty759. 30169615

[ref42] Tang J.; Szwajda A.; Shakyawar S.; Xu T.; Hintsanen P.; Wennerberg K.; Aittokallio T. Making Sense of Large-Scale Kinase Inhibitor Bioactivity Data Sets: A Comparative and Integrative Analysis. J. Chem. Inf. Model. 2014, 54, 735–743. 10.1021/ci400709d. 24521231

[ref43] Lim J.; Ryu S.; Kim J. W.; Kim W. Y. Molecular Generative Model Based on Conditional Variational Autoencoder for De Novo Molecular Design. Aust. J. Chem. 2018, 10, 31 10.1186/s13321-018-0286-7. PMC6041224 29995272

[ref44] Pires D. E. V.; Ascher D. B. Mcsm-Ab: A Web Server for Predicting Antibody-Antigen Affinity Changes Upon Mutation with Graph-Based Signatures. Nucleic Acids Res. 2016, 44, W469–W473. 10.1093/nar/gkw458. 27216816 PMC4987957

[ref45] Rodrigues C. H. M.; Pires D. E. V.; Ascher D. B. Dynamut2: Assessing Changes in Stability and Flexibility Upon Single and Multiple Point Missense Mutations. Protein Sci. 2021, 30, 60–69. 10.1002/pro.3942. 32881105 PMC7737773

[ref46] Myung Y.; Rodrigues C. H. M.; Ascher D. B.; Pires D. E. V. Mcsm-Ab2: Guiding Rational Antibody Design Using Graph-Based Signatures. Bioinformatics 2020. 10.1093/bioinformatics/btz779 31665262

[ref47] Pires D. E. V.; Blundell T. L.; Ascher D. B. Mcsm-Lig: Quantifying the Effects of Mutations on Protein-Small Molecule Affinity in Genetic Disease and Emergence of Drug Resistance. Sci. Rep. 2016, 6, 29575 10.1038/srep29575. 27384129 PMC4935856

[ref48] Pires D. E. V.; Ascher D. B. Mcsm-Na: Predicting the Effects of Mutations on Protein-Nucleic Acids Interactions. Nucleic Acids Res. 2017, W241 10.1093/nar/gkx236. 28383703 PMC5570212

[ref49] Rodrigues C. H.; Pires D. E.; Ascher D. B. Dynamut: Predicting the Impact of Mutations on Protein Conformation, Flexibility and Stability. Nucleic Acids Res. 2018, 46, W350–W355. 10.1093/nar/gky300. 29718330 PMC6031064

[ref50] Landrum G. Rdkit: Open-Source Cheminformatics. 2006.

[ref51] Pires D. E.; de Melo-Minardi R. C.; dos Santos M. A.; da Silveira C. H.; Santoro M. M.; Meira W. Jr. Cutoff Scanning Matrix (Csm): Structural Classification and Function Prediction by Protein Inter-Residue Distance Patterns. BMC Genomics 2011, 12, S12 10.1186/1471-2164-12-S4-S12. PMC3287581 22369665

[ref52] Zhang Z.; Guan J.; Zhou S. Fragat: A Fragment-Oriented Multi-Scale Graph Attention Model for Molecular Property Prediction. Bioinformatics 2021, btab195 10.1093/bioinformatics/btab195. 33769437 PMC8479684

[ref53] Li H.; Phung D. Journal of Machine Learning Research: Preface. J. Mach. Learn. Res. 2014, 39, i–ii.

[ref54] Riddick G.; Song H.; Ahn S.; Walling J.; Borges-Rivera D.; Zhang W.; Fine H. A. Predicting in Vitro Drug Sensitivity Using Random Forests. Bioinformatics 2011, 220 10.1093/bioinformatics/btq628. PMC3018816 21134890

[ref55] Müller A. C.; Guido S. Introduction to Machine Learning with Python; O’Reilly Media. 2017; p 121–130.

