# Supplementary material for: pdCSM-cancer: Using Graph-Based Signatures to Identify Small Molecules with Anticancer Properties
Source: J Chem Inf Model. 2021 Jul 2;61(7):3314–22. doi: 10.1021/acs.jcim.1c00168 (PMC8317153; doi:10.1021/acs.jcim.1c00168)
Supplement: Supplementary file 1 — ci1c00168_si_001.pdf [file ci1c00168_si_001.pdf]

SUPPORTING INFORMATION

pdCSM-cancer: Using Graph-Based Signatures to Identify Small Molecules with Anticancer Properties

Raghad Al-Jarf[1,2,3], Alex G.C. de Sá[1,2,3,4], Douglas E.V. Pires[1,2,3,5*], David B. Ascher[1,2,3,4,6*]

[1]Structural Biology and Bioinformatics, Department of Biochemistry, University of Melbourne, Parkville 3052, Victoria, Australia
[2]Systems and Computational Biology, Bio21 Institute, University of Melbourne, Parkville 3052, Victoria, Australia
[3]Computational Biology and Clinical Informatics, Baker Heart and Diabetes Institute, Melbourne 3004, Victoria, Australia
[4]Baker Department of Cardiometabolic Health, Melbourne Medical School, University of Melbourne, Parkville 3010,Victoria, Australia
[5]School of Computing and Information Systems, University of Melbourne, Parkville 3052, Victoria, Australia
[6]Department of Biochemistry, University of Cambridge, 80 Tennis Ct Rd, Cambridge CB2 1GA

*To whom correspondence should be addressed D.B.A. Tel: +61 90354794; Email: david.ascher@unimelb.edu.au.
Correspondence may also be addressed to D.E.V.P. douglas.pires@unimelb.edu.au.

FIGURES

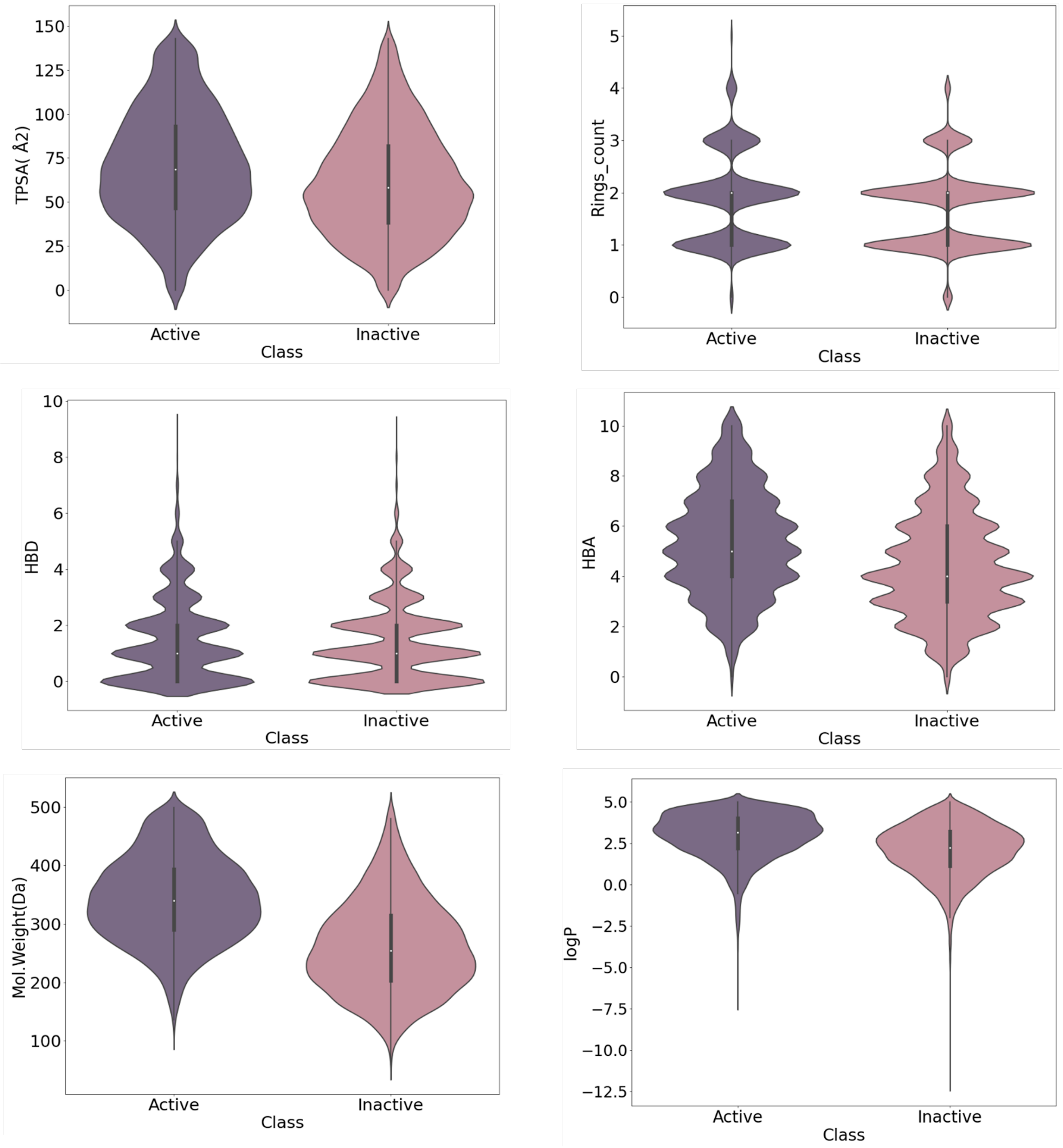

Figure S1. Physiochemical properties of compounds with anticancer activity. The violin plots depict the physicochemical properties of the molecules, including ring count, Molecular weight, hydrogen acceptor and donor counts, log P, and topological polar surface area (TPSA). Both active and inactive compounds obey Lipinski's rule of five (RO5).

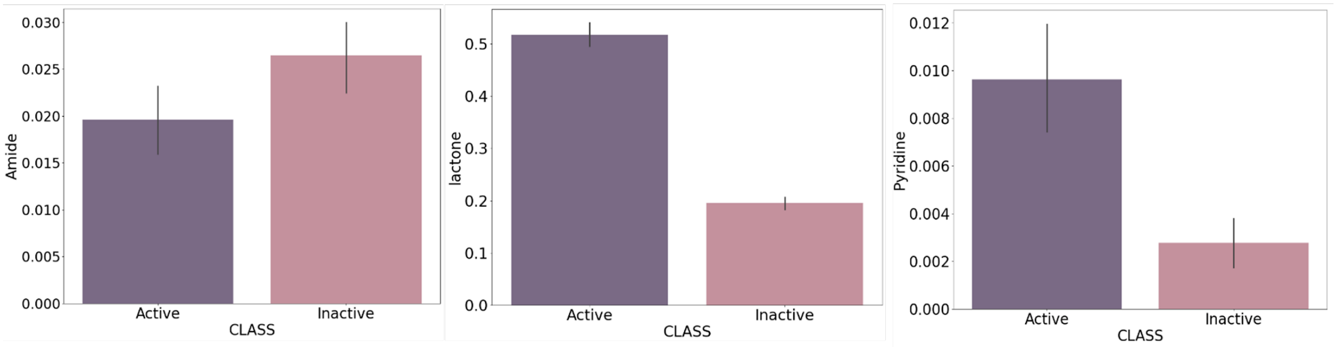

Figure S2. Property distributions of active compounds compared to inactive. The plots depict the top discriminative features of active molecules in comparison to the inactive molecules. Molecules with anticancer activity tended to have a high frequency of lactone (middle) and pyridine (right), with p-values < 1.9 e-07. In contrast, the inactive compounds tended to have a higher frequency of amide (left).

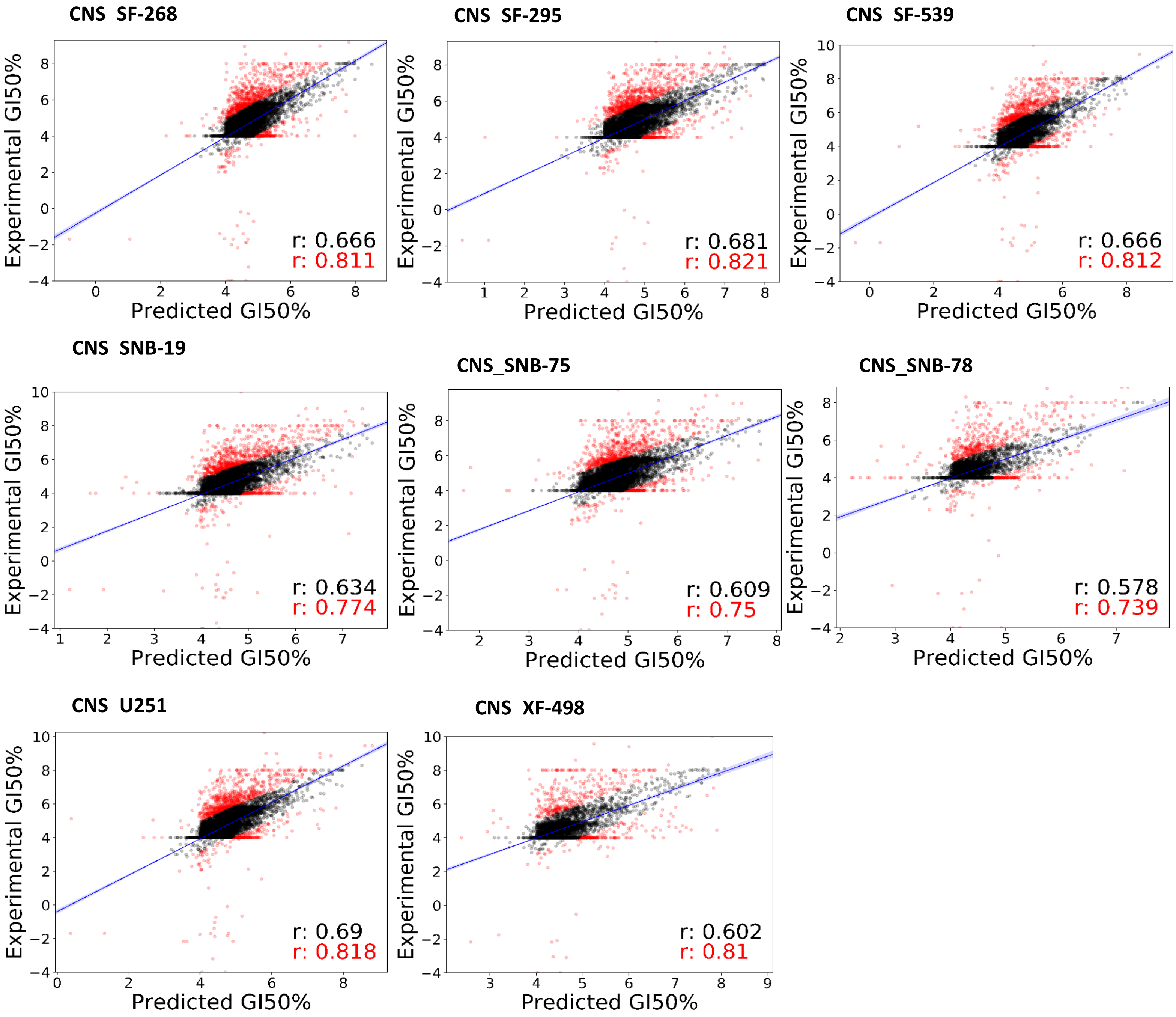

Figure S3. Performance of pdCSM-cancer (CNS panel) under 10-fold cross-validation. Scatter plots between experimental and predicted GI50% values given in -log 10(molar) for each of the cell line models of the CNS panel are displayed. Pearson's correlation coefficient (r) is shown for each scatter plot (in black for 100% of the data and red for 90% of the data, after 10% outlier removal).

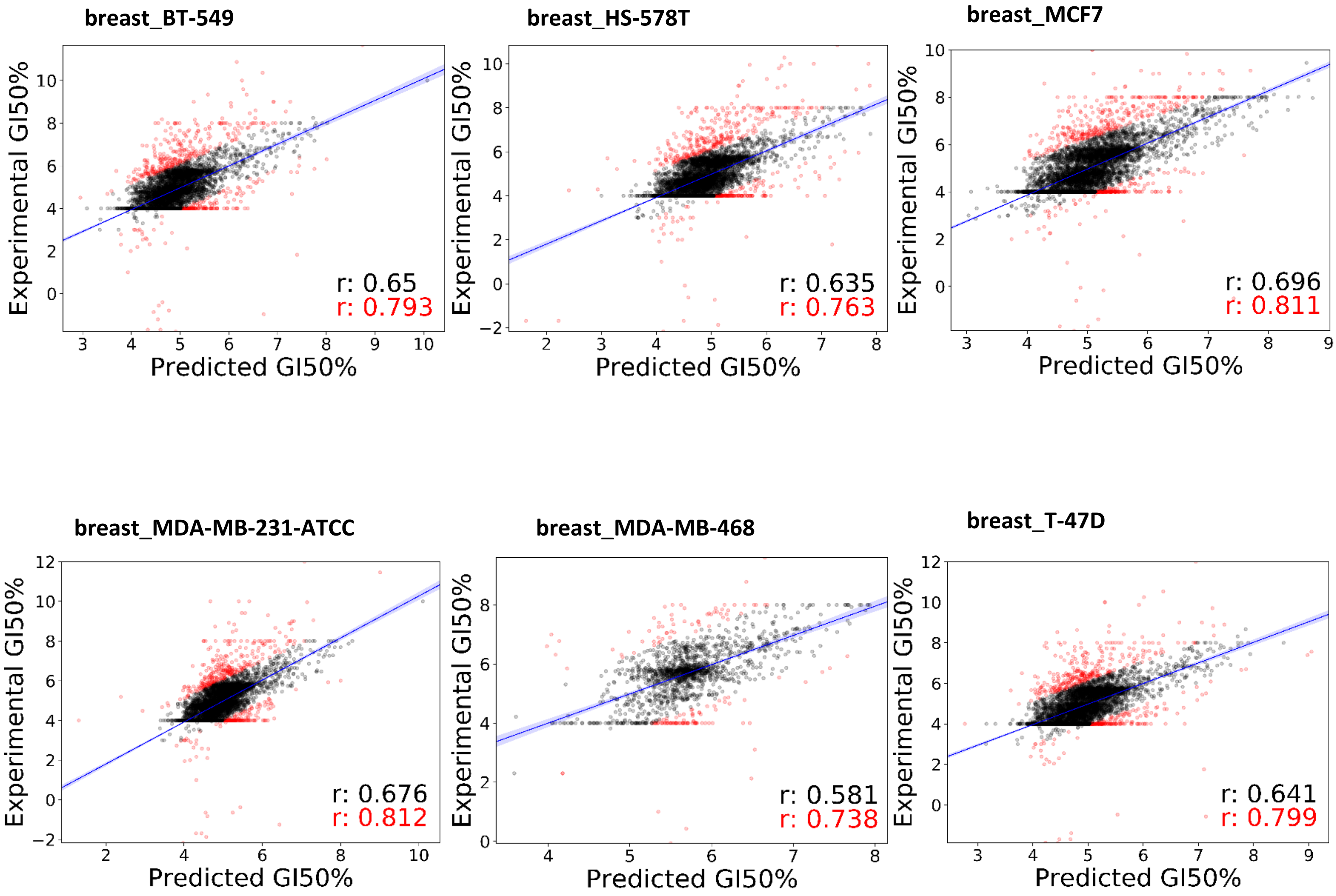

Figure S4. Performance of pdCSM-cancer (Breast cancer panel) under 10-fold cross-validation. Scatter plots between experimental and predicted GI50% values given in -log 10(molar) for each of the cell line models of the breast panel are displayed. Pearson's correlation coefficient (r) is shown for each scatter plot (in black for 100% of the data and red for 90% of the data, after 10% outlier removal).

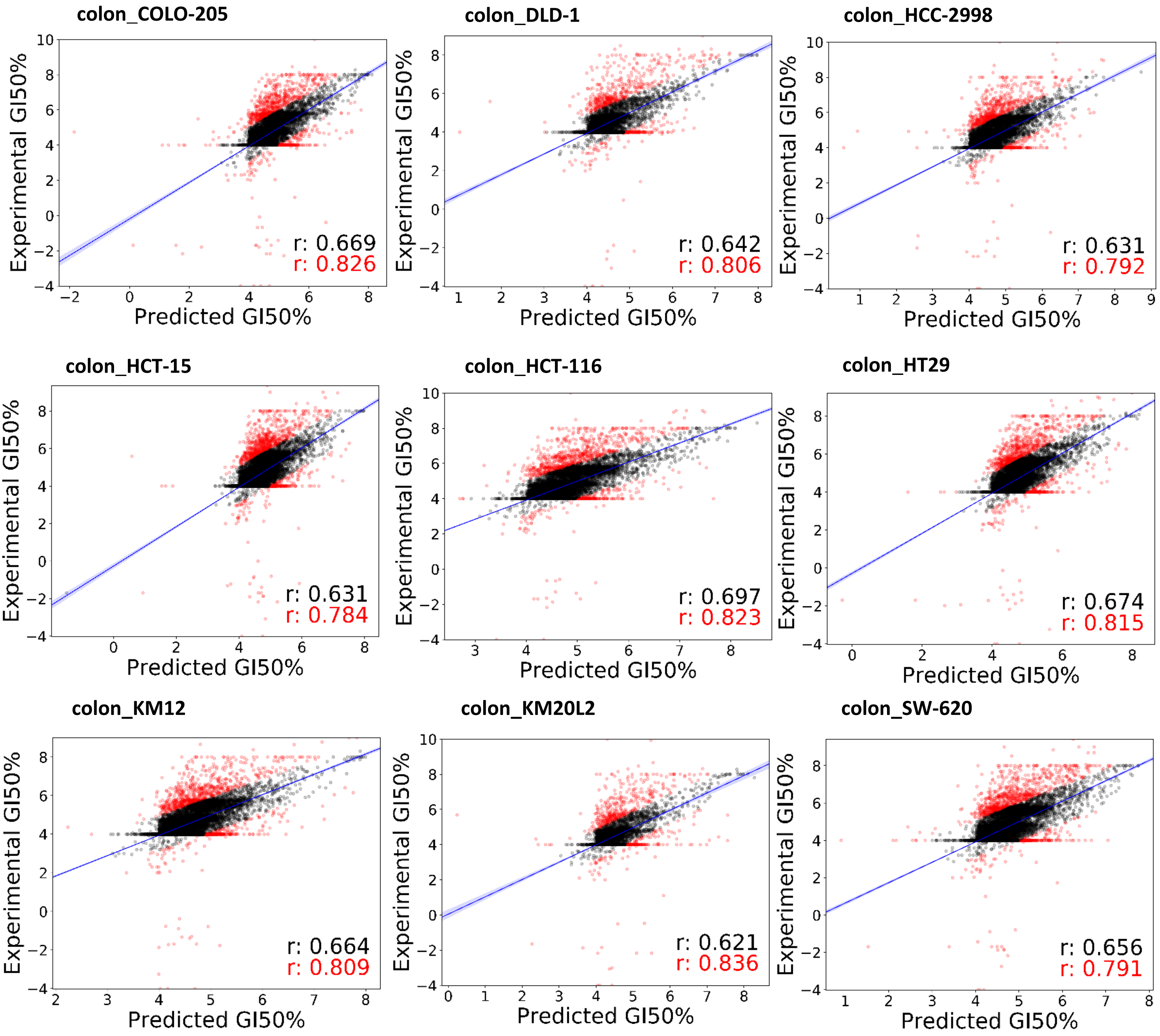

Figure S5. Performance of pdCSM-cancer (Colon cancer panel) under 10-fold cross-validation. Scatter plots between experimental and predicted GI50% values given in -log 10(molar) for each of the cell line models of the colon panel are displayed. Pearson's correlation coefficient (r) is shown for each scatter plot (in black for 100% of the data and red for 90% of the data, after 10% outlier removal).

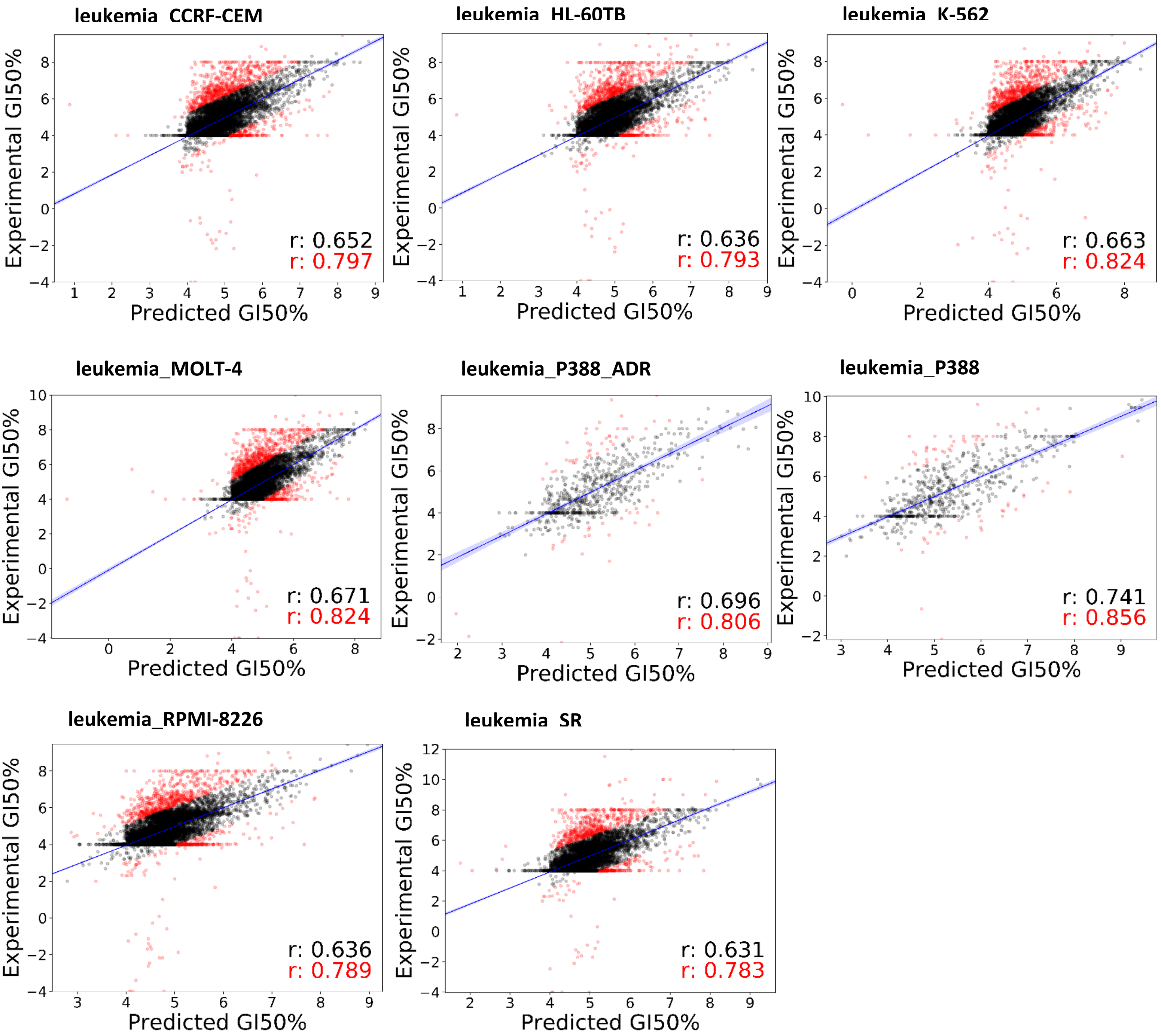

Figure S6. Performance of pdCSM-cancer (Leukemia panel) under 10-fold cross-validation. Scatter plots between experimental and predicted GI50% values given in -log 10(molar) for each of the cell line models of the Leukemia panel are displayed. Pearson's correlation coefficient (r) is shown for each scatter plot (in black for 100% of the data and red for 90% of the data, after 10% outlier removal).

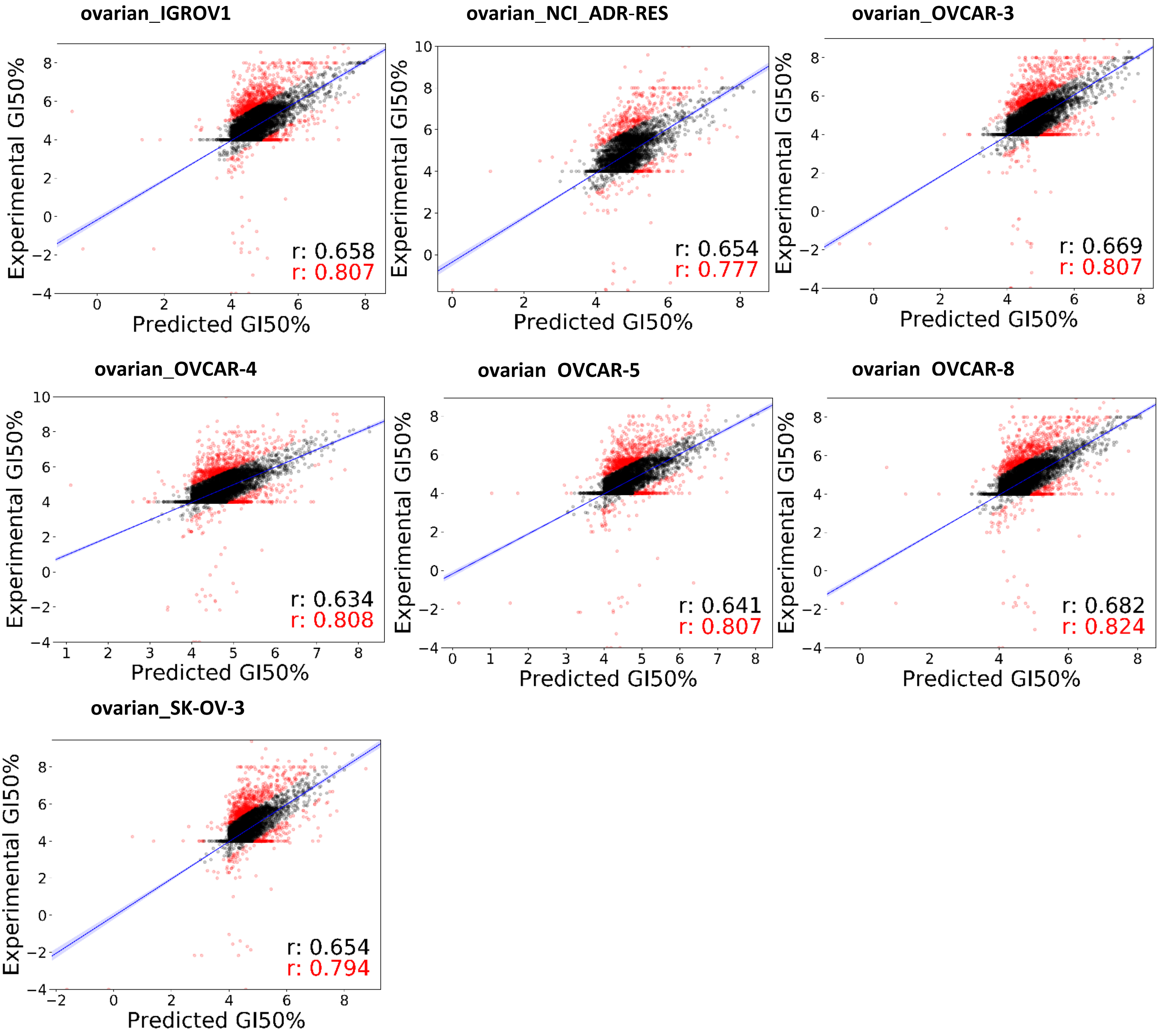

Figure S7. Performance of pdCSM-cancer (Ovarian cancer panel) under 10-fold cross-validation. Scatter plots between experimental and predicted GI50% values given in -log 10(molar) for each of the cell line models of the Ovarian panel are displayed. Pearson's correlation coefficient (r) is shown for each scatter plot (in black for 100% of the data and red for 90% of the data, after 10% outlier removal).

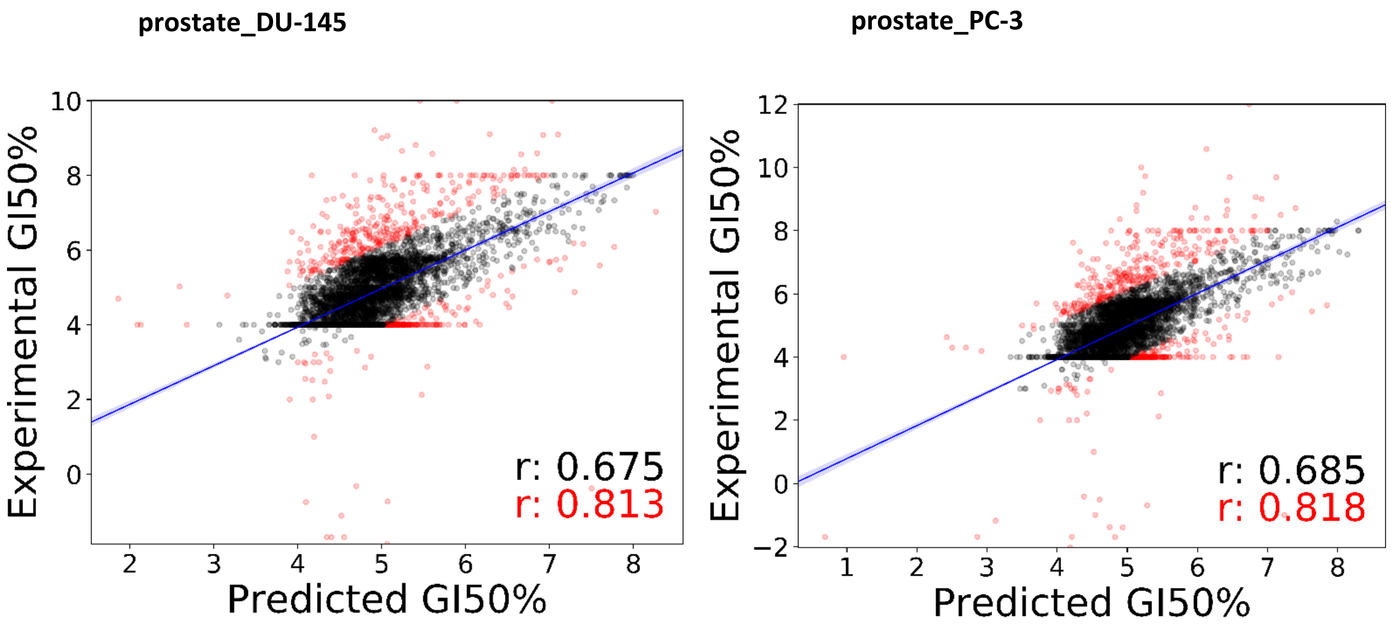

Figure S8. Performance of pdCSM-cancer (Prostate cancer panel) under 10-fold cross-validation. Scatter plots between experimental and predicted GI50% values given in -log 10(molar) for each of the cell line models of the Prostate panel are displayed. Pearson's correlation coefficient (r) is shown for each scatter plot (in black for 100% of the data and red for 90% of the data, after 10% outlier removal).

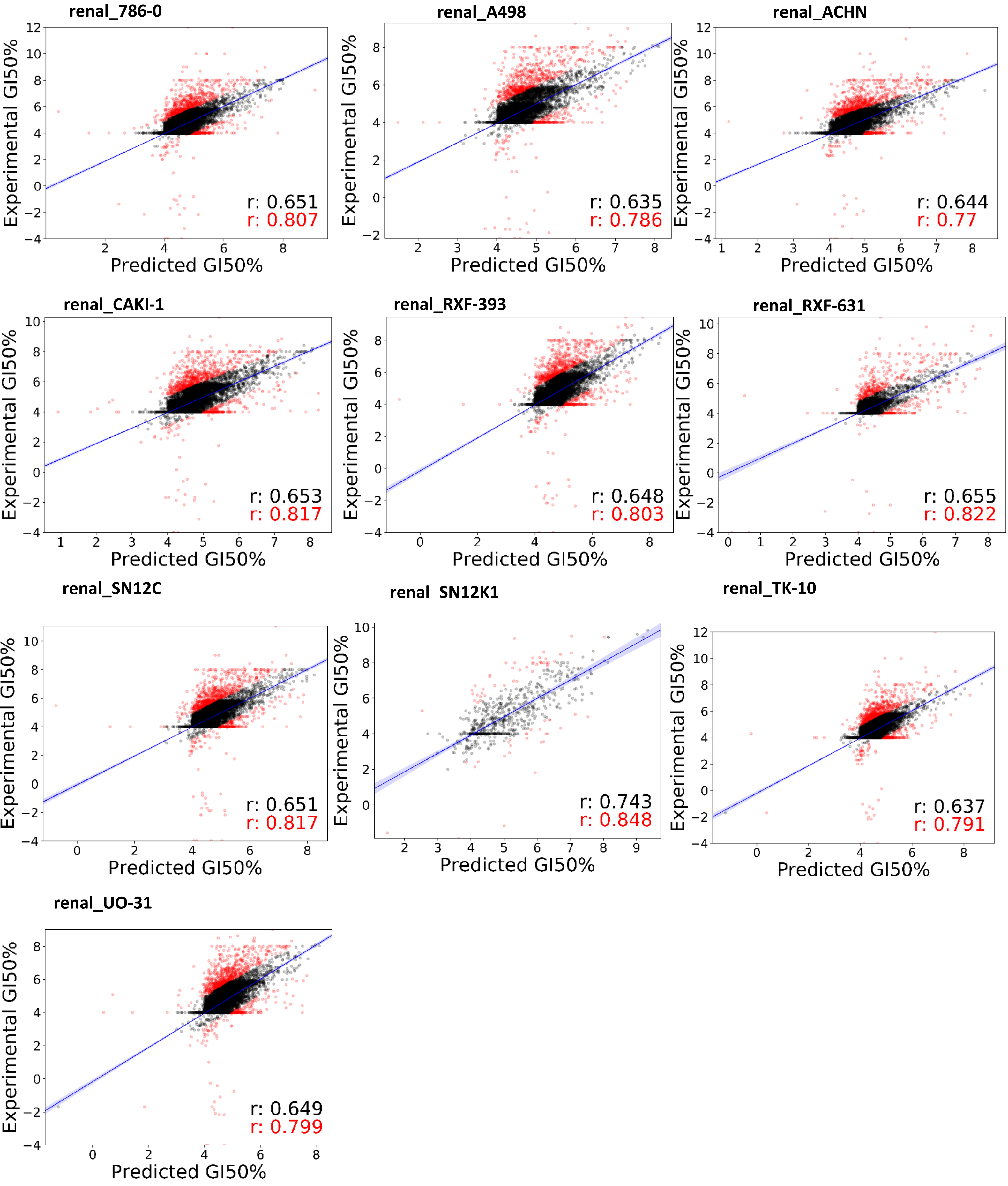

Figure S9. Performance of pdCSM-cancer (Renal cancer panel) under 10-fold cross-validation. Scatter plots between experimental and predicted GI50% values given in -log 10(molar) for each of the cell line models of the Renal panel are displayed. Pearson's correlation coefficient (r) is shown for each scatter plot (in black for 100% of the data and red for 90% of the data, after 10% outlier removal).

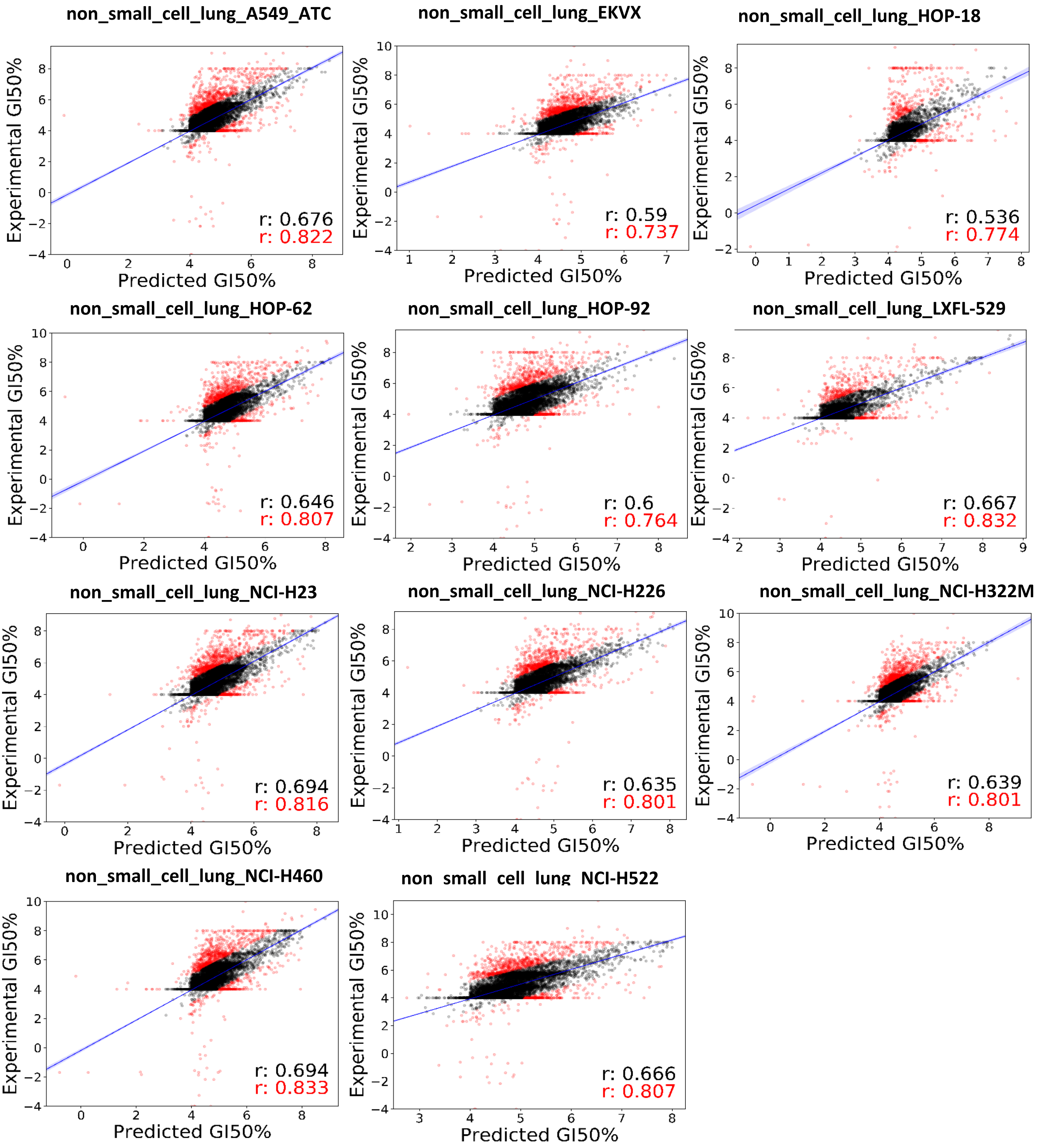

Figure S10. Performance of pdCSM-cancer (Non-small cell lung cancer panel) under 10-fold cross-validation. Scatter plots between experimental and predicted GI50% values given in -log 10(molar) for each of the cell line models of the Non-small cell lung panel are displayed. Pearson's correlation coefficient (r) is shown for each scatter plot (in black for 100% of the data and red for 90% of the data, after 10% outlier removal).

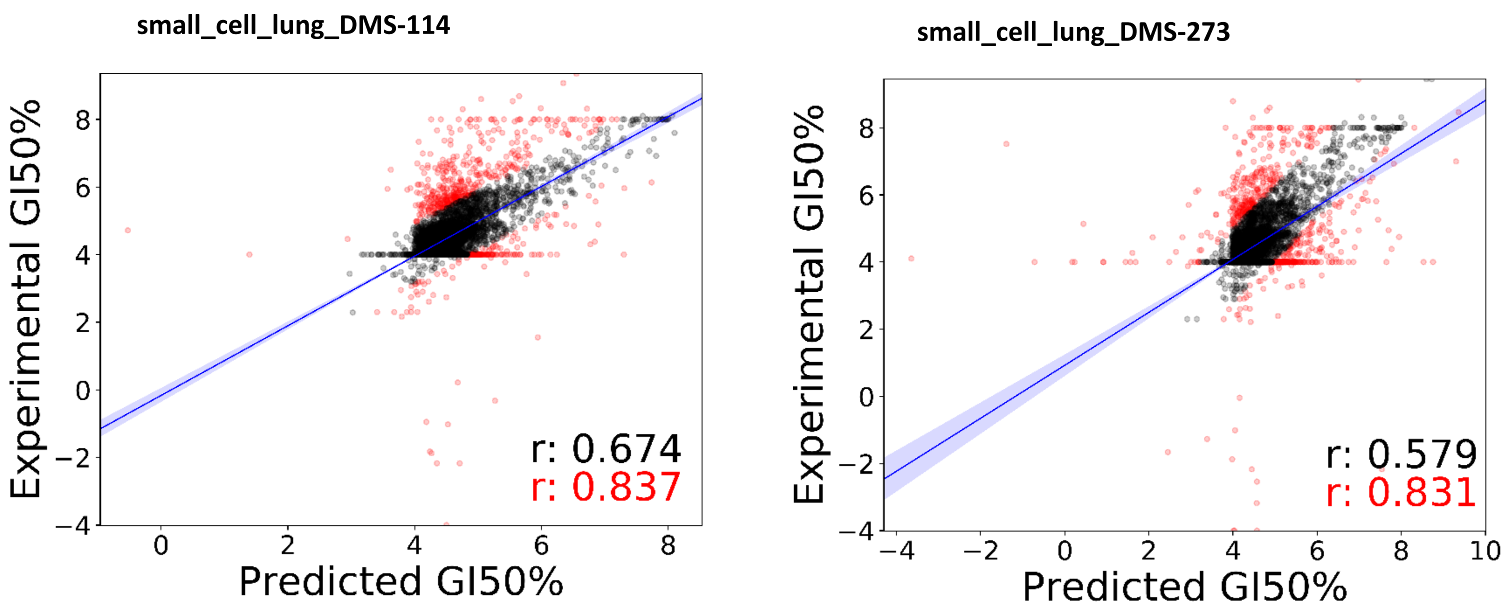

Figure S11. Performance of pdCSM-cancer (Small cell lung cancer panel) under 10-fold cross-validation. Scatter plots between experimental and predicted GI50% values given in -log 10(molar) for each of the cell line models of the small cell lung panel are displayed. Pearson's correlation coefficient (r) is shown for each scatter plot (in black for 100% of the data and red for 90% of the data, after 10% outlier removal).

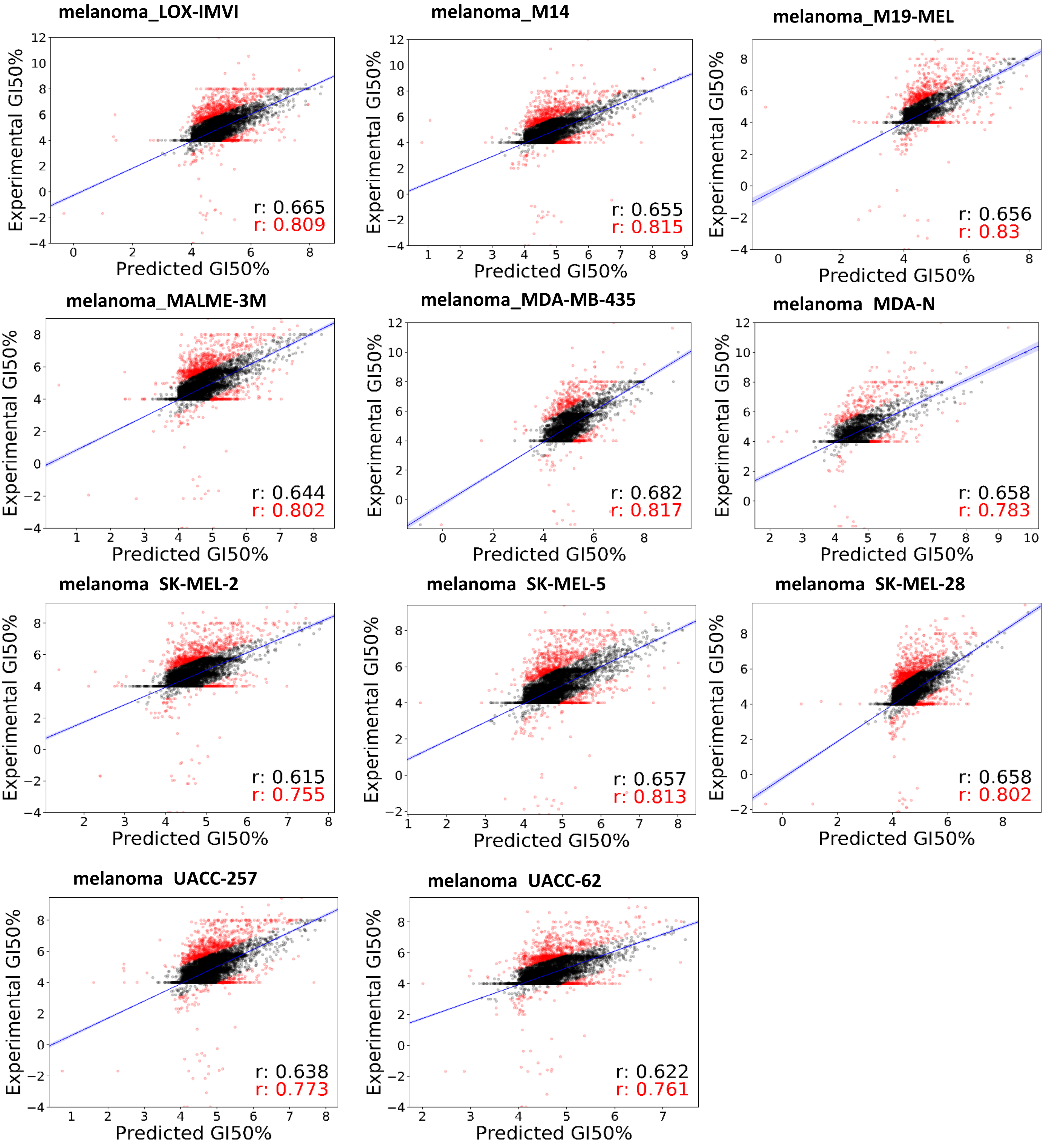

Figure S12. Performance of pdCSM-cancer (Melanoma panel) under 10-fold cross-validation. Scatter plots between experimental and predicted GI50% values given in -log 10(molar) for each of the cell line models of the Melanoma panel are displayed. Pearson's correlation coefficient (r) is shown for each scatter plot (in black for 100% of the data and red for 90% of the data, after 10% outlier removal).

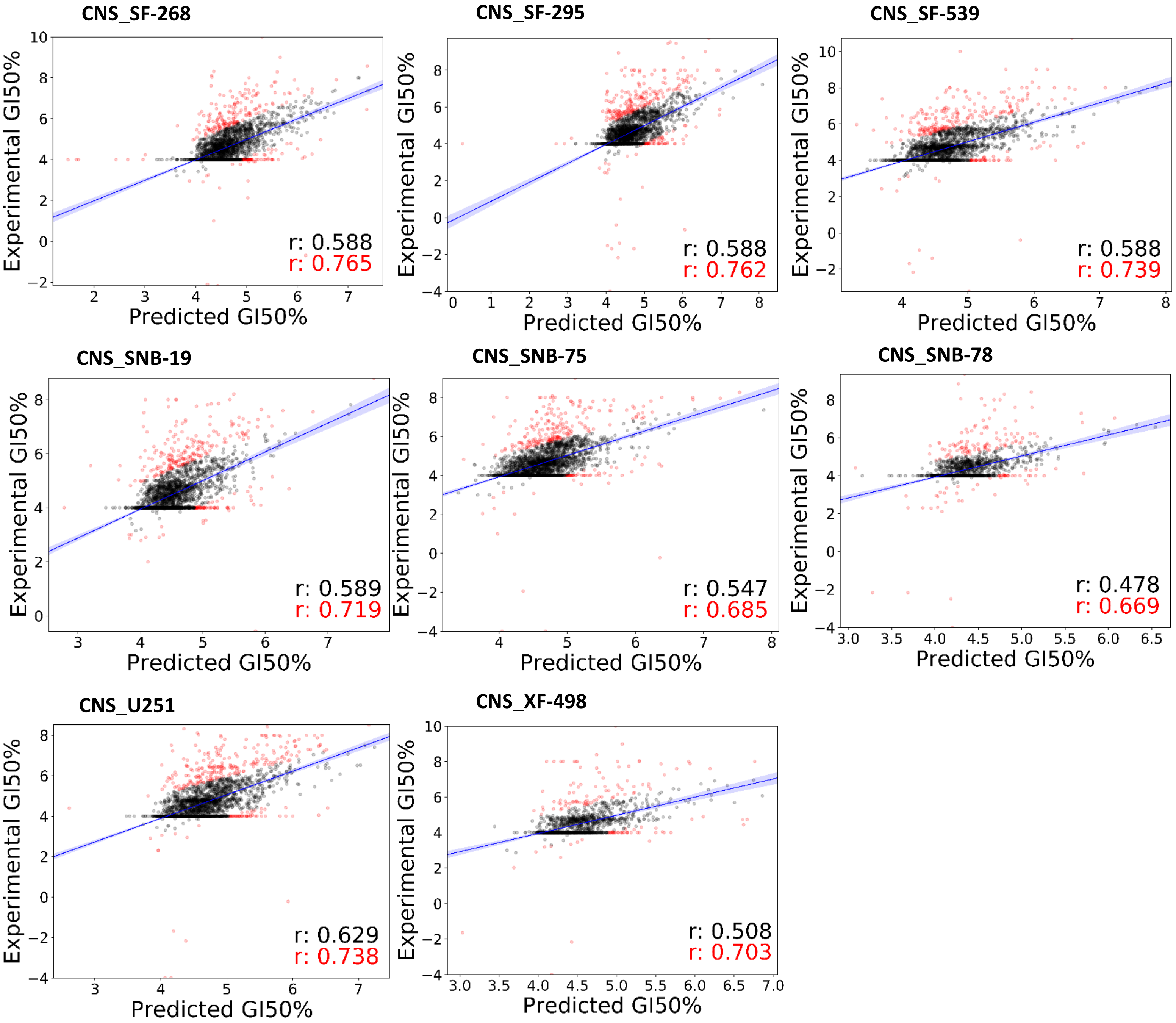

Figure S13. Performance of pdCSM-cancer (CNS panel) on independent blind tests. Scatter plots between experimental and predicted GI50% values given in -log 10(molar) for each of the cell line models of the CNS panel are displayed. Pearson's correlation coefficient (r) is shown for each scatter plot (in black for 100% of the data and red for 90% of the data, after 10% outlier removal).

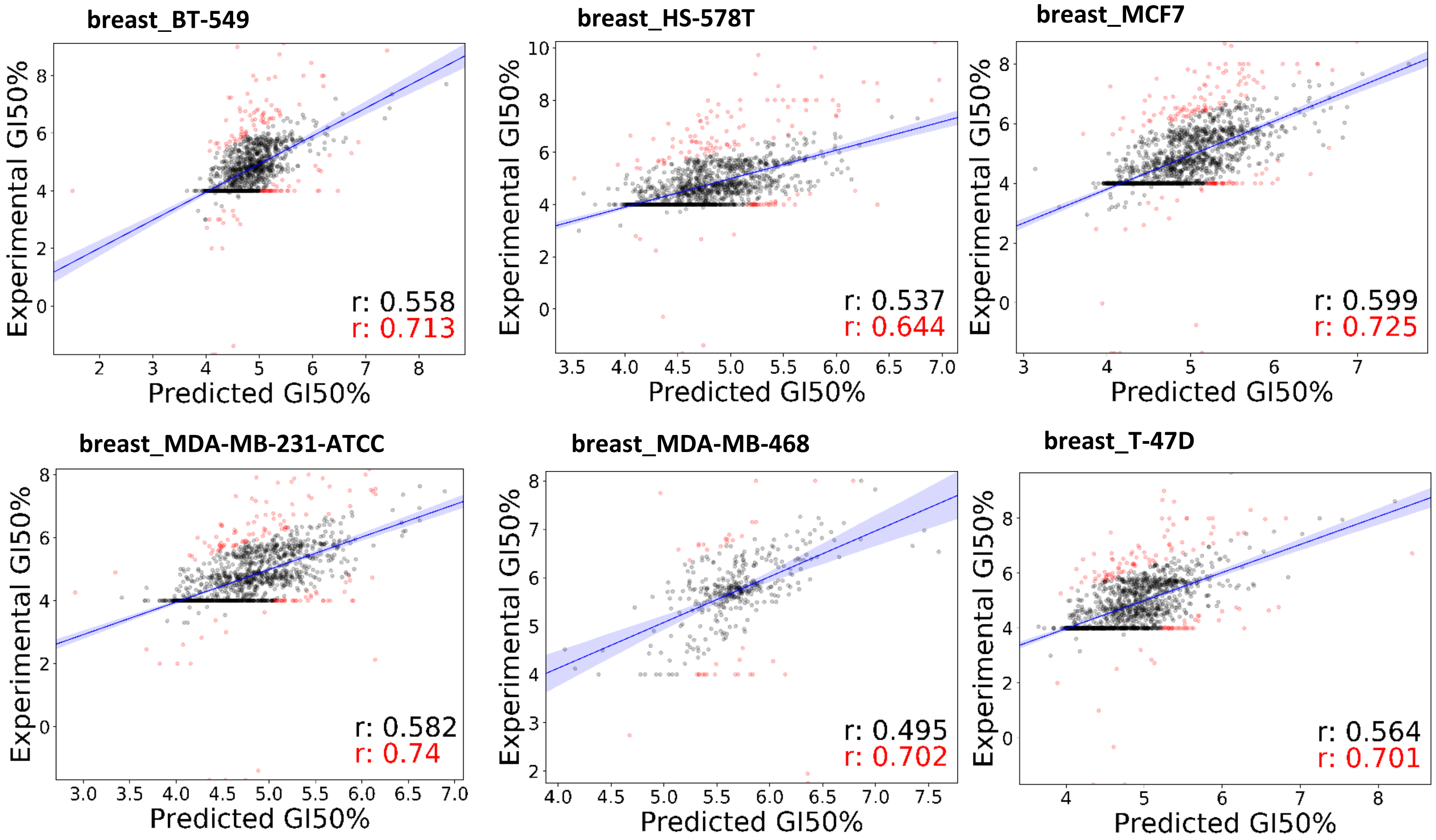

Figure S14. Performance of pdCSM-cancer (Breast cancer panel) on independent blind tests. Scatter plots between experimental and predicted GI50% values given in -log 10(molar) for each of the cell line models of the breast panel are displayed. Pearson's correlation coefficient (r) is shown for each scatter plot (in black for 100% of the data and red for 90% of the data, after 10% outlier removal).

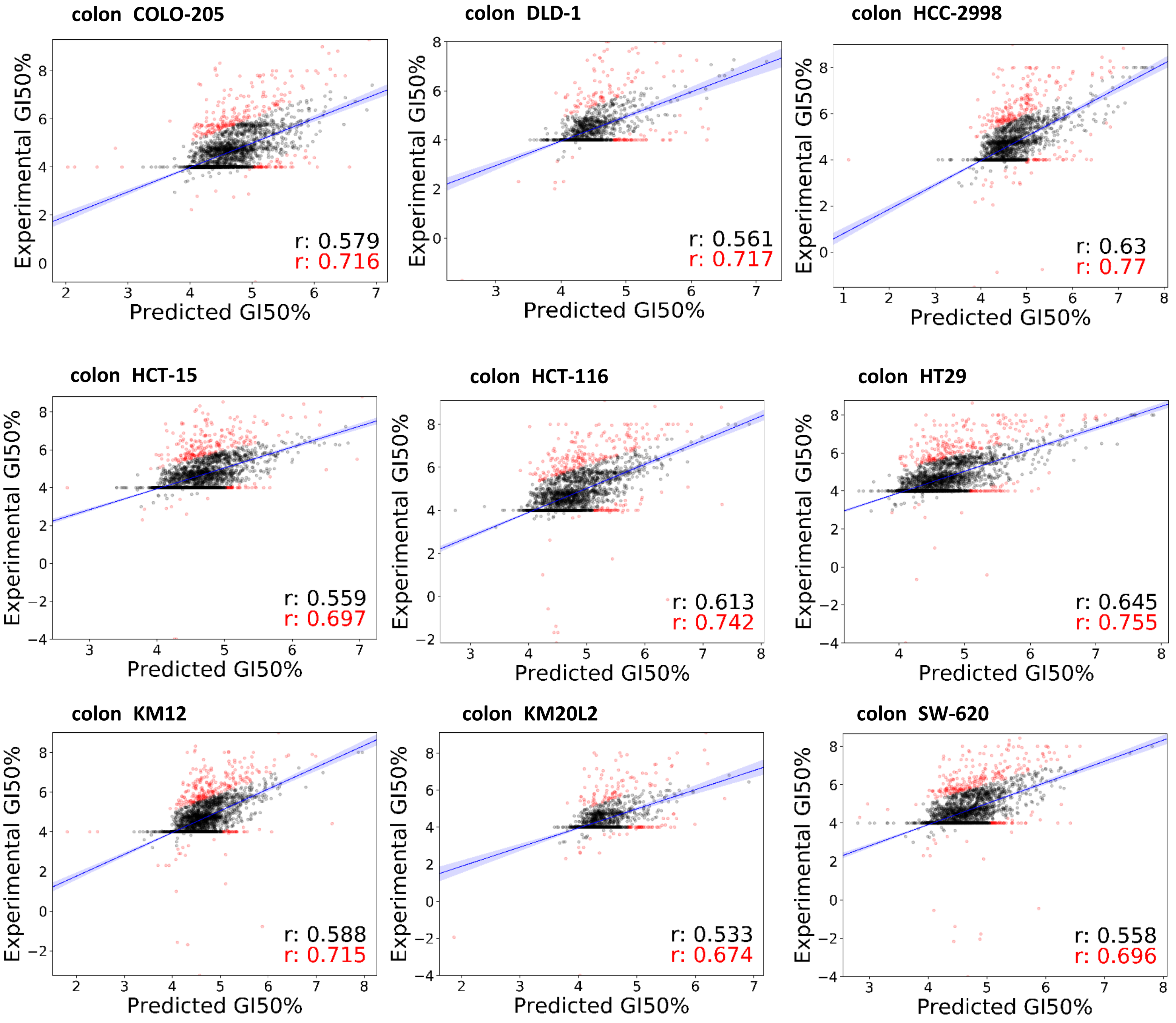

Figure S15. Performance of pdCSM-cancer (Colon cancer panel) on independent blind tests. Scatter plots between experimental and predicted GI50% values given in -log 10(molar) for each of the cell line models of the colon cancer panel are displayed. Pearson's correlation coefficient (r) is shown for each scatter plot (in black for 100% of the data and red for 90% of the data, after 10% outlier removal).

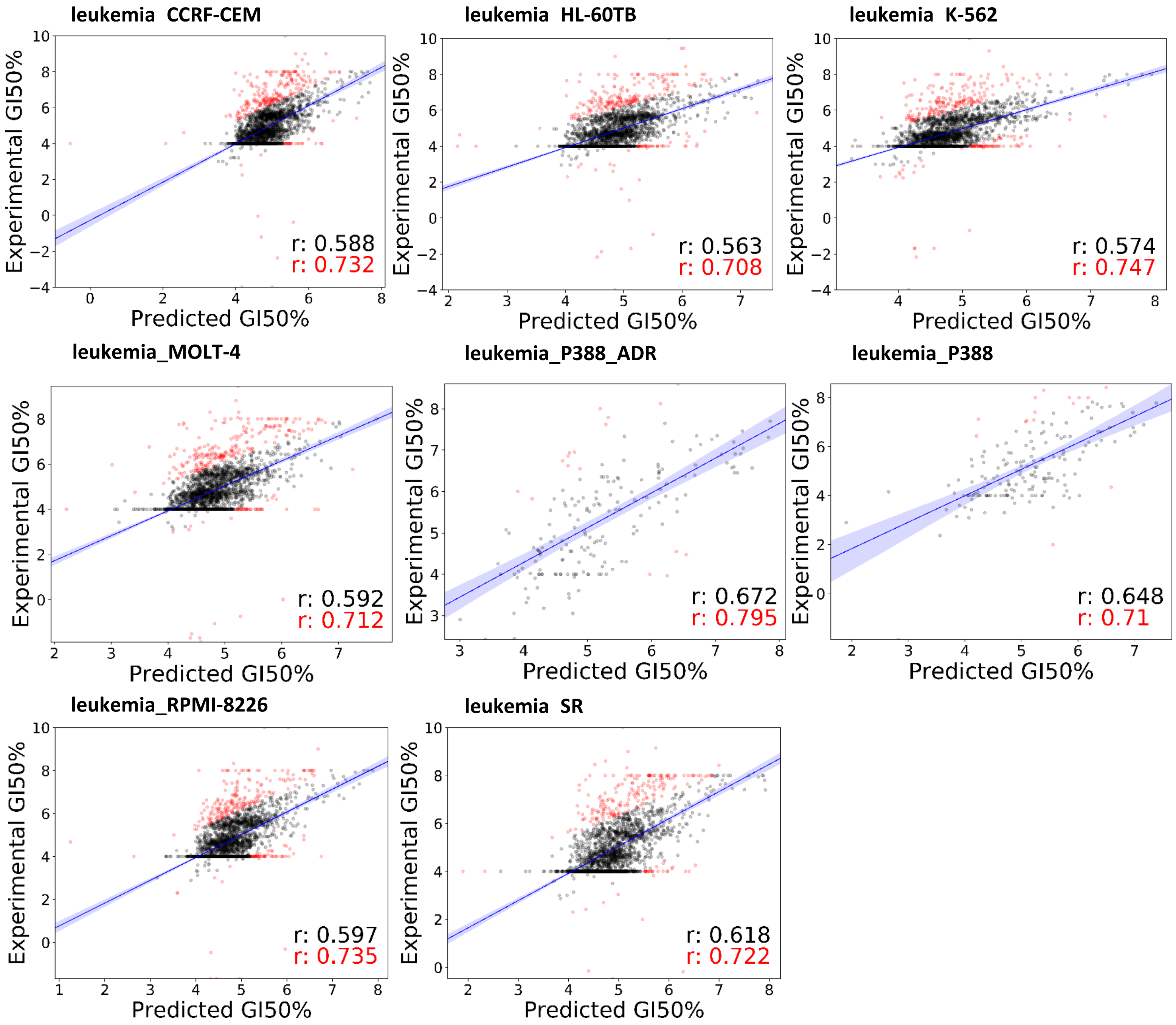

Figure S16. Performance of pdCSM-cancer (Leukemia panel) on independent blind tests. Scatter plots between experimental and predicted GI50% values given in -log 10(molar) for each of the cell line models of the Leukemia panel are displayed. Pearson's correlation coefficient (r) is shown for each scatter plot (in black for 100% of the data and red for 90% of the data, after 10% outlier removal).

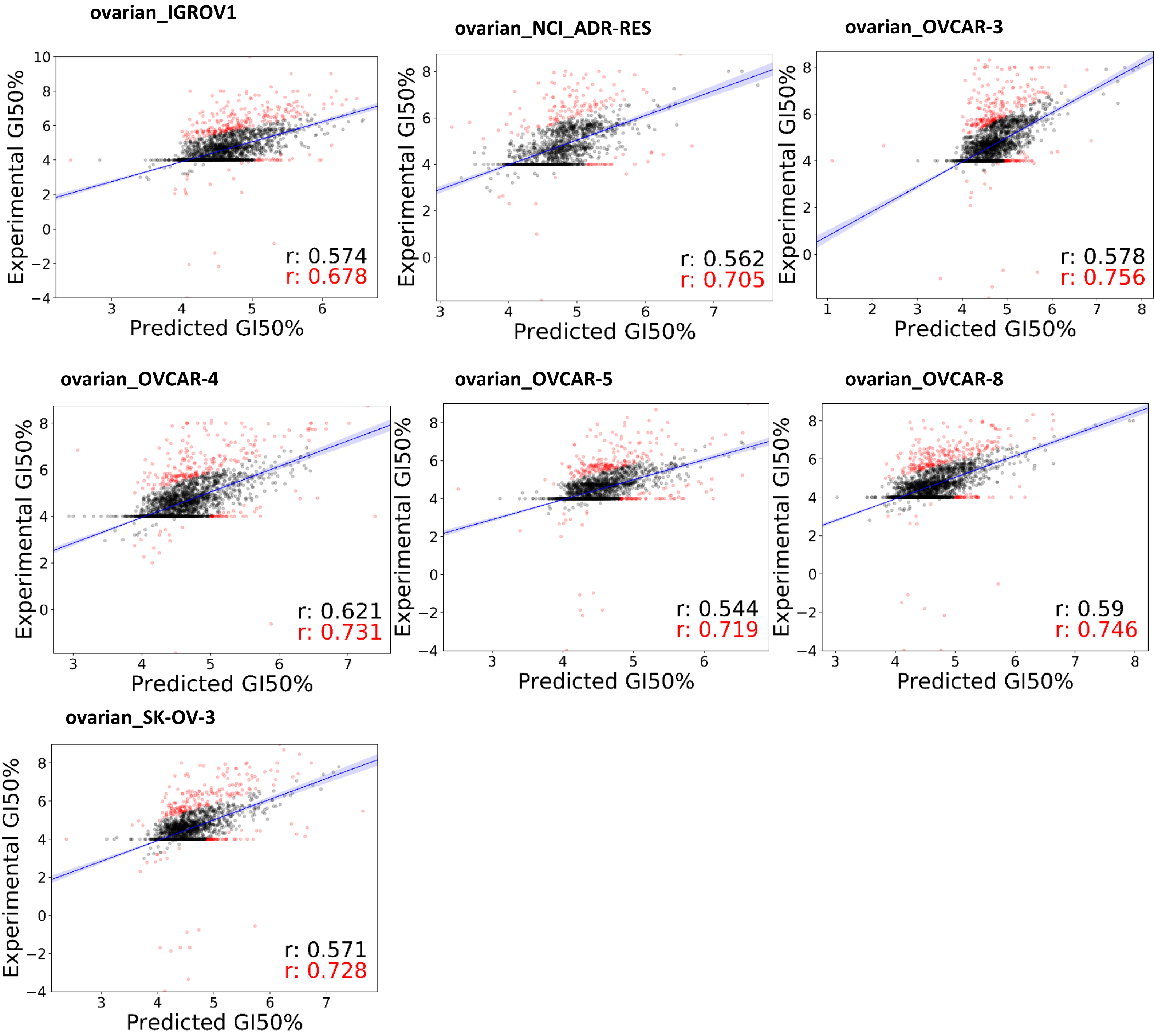

Figure S17. Performance of pdCSM-cancer (Ovarian cancer panel) on independent blind tests. Scatter plots between experimental and predicted GI50% values given in -log 10(molar) for each of the cell line models of the Leukemia panel are displayed. Pearson's correlation coefficient (r) is shown for each scatter plot (in black for 100% of the data and red for 90% of the data, after 10% outlier removal).

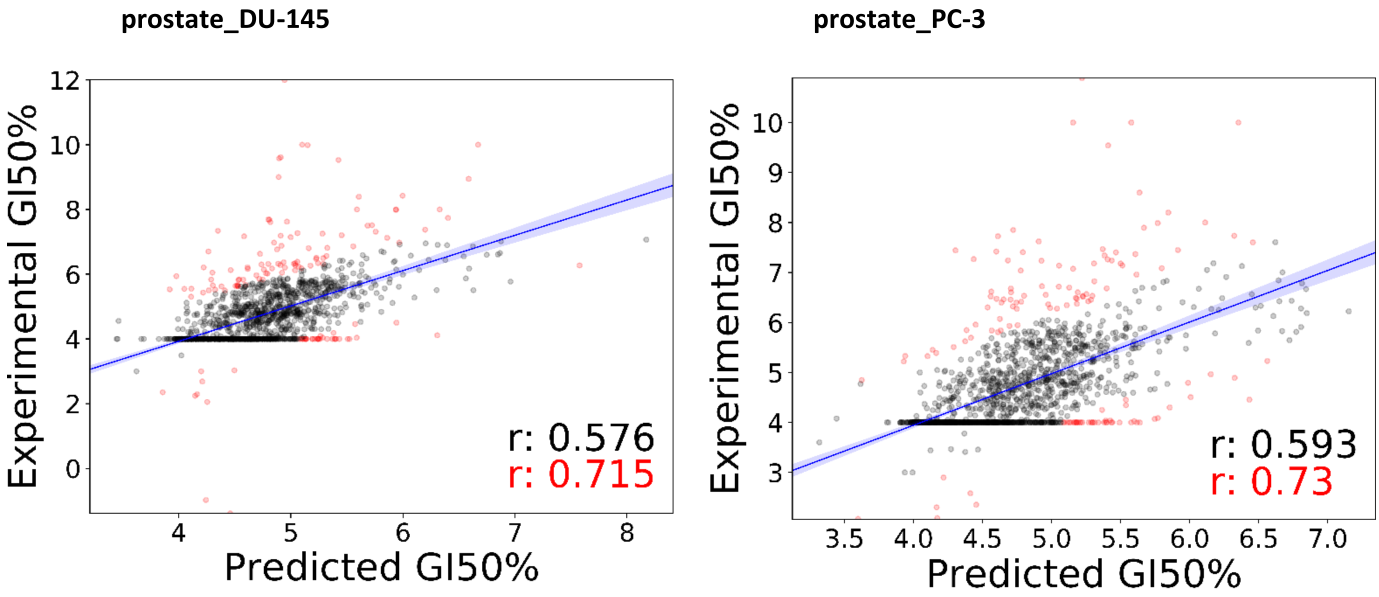

Figure S18. Performance of pdCSM-cancer (Prostate cancer panel) on independent blind tests. Scatter plots between experimental and predicted GI50% values given in -log 10(molar) for each of the cell line models of the Prostate panel are displayed. Pearson's correlation coefficient (r) is shown for each scatter plot (in black for 100% of the data and red for 90% of the data, after 10% outlier removal).

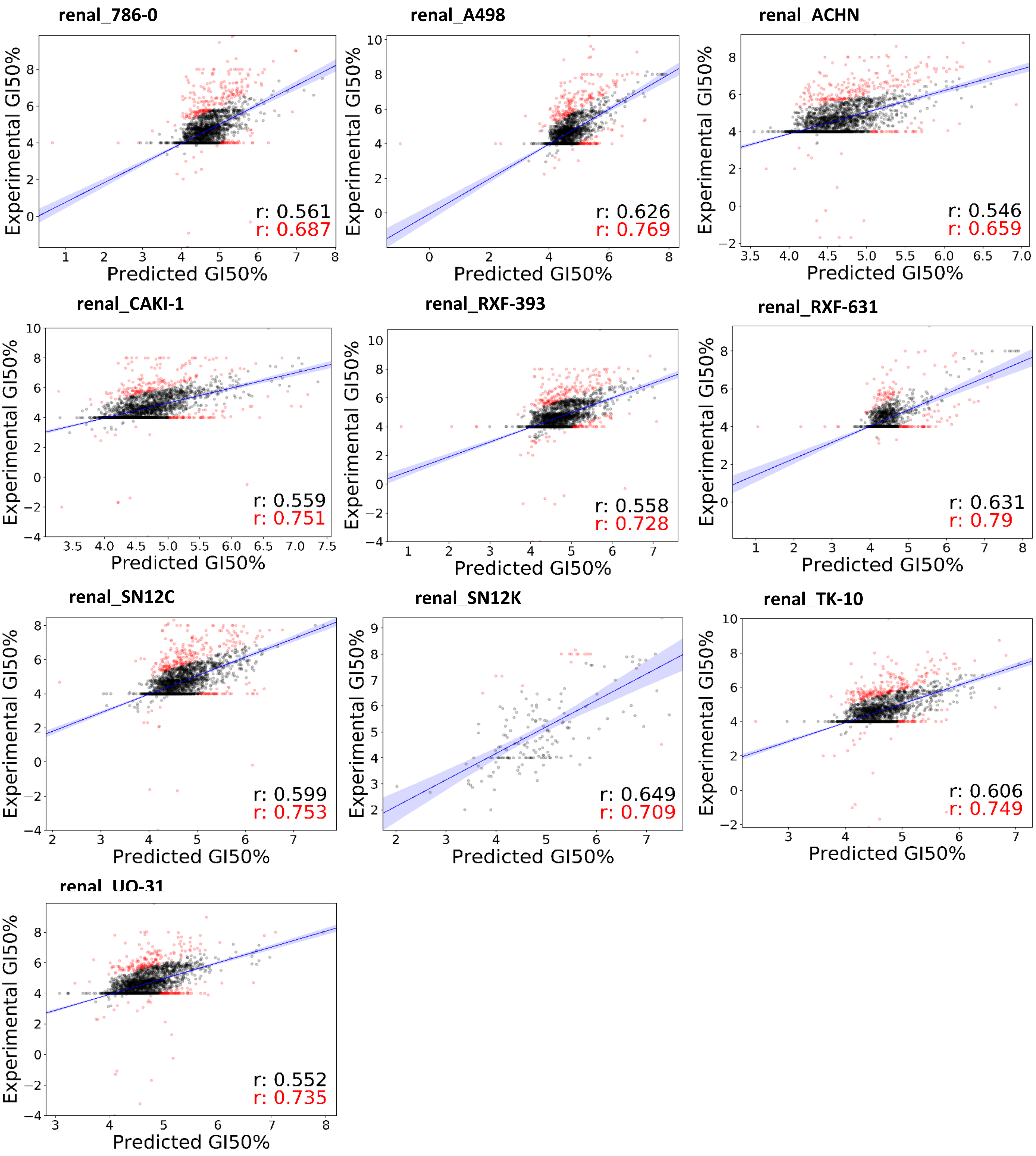

Figure S19. Performance of pdCSM-cancer (Renal cancer panel) on independent blind tests. Scatter plots between experimental and predicted GI50% values given in -log 10(molar) for each of the cell line models of the Renal panel are displayed. Pearson's correlation coefficient (r) is shown for each scatter plot (in black for 100% of the data and red for 90% of the data, after 10% outlier removal).

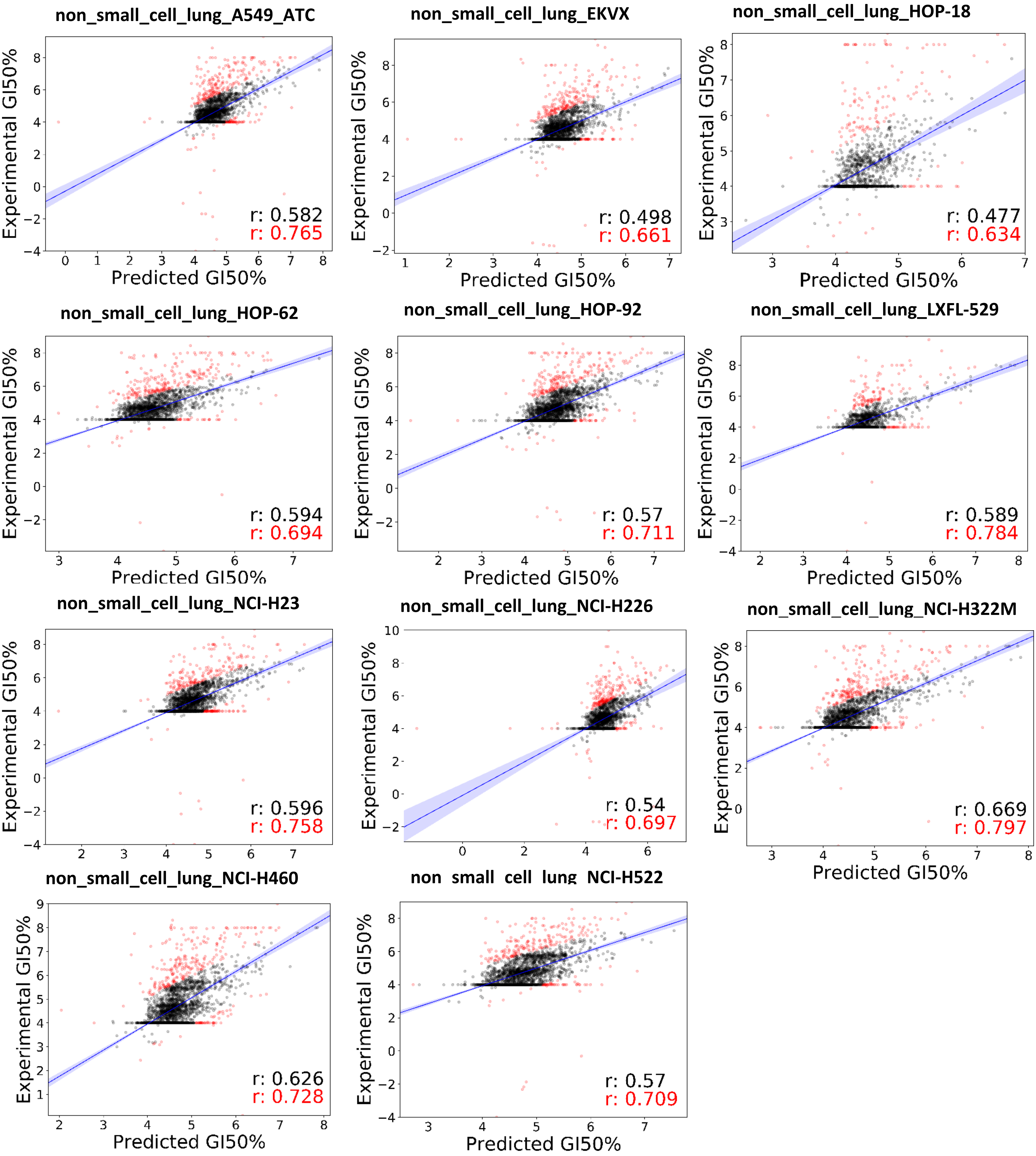

Figure S20. Performance of pdCSM-cancer (Non-small cell lung cancer panel) on independent blind tests. Scatter plots between experimental and predicted GI50% values given in -log 10(molar) for each of the cell line models of the Non-small cell lung panel are displayed. Pearson's correlation coefficient (r) is shown for each scatter plot (in black for 100% of the data and red for 90% of the data, after 10% outlier removal).

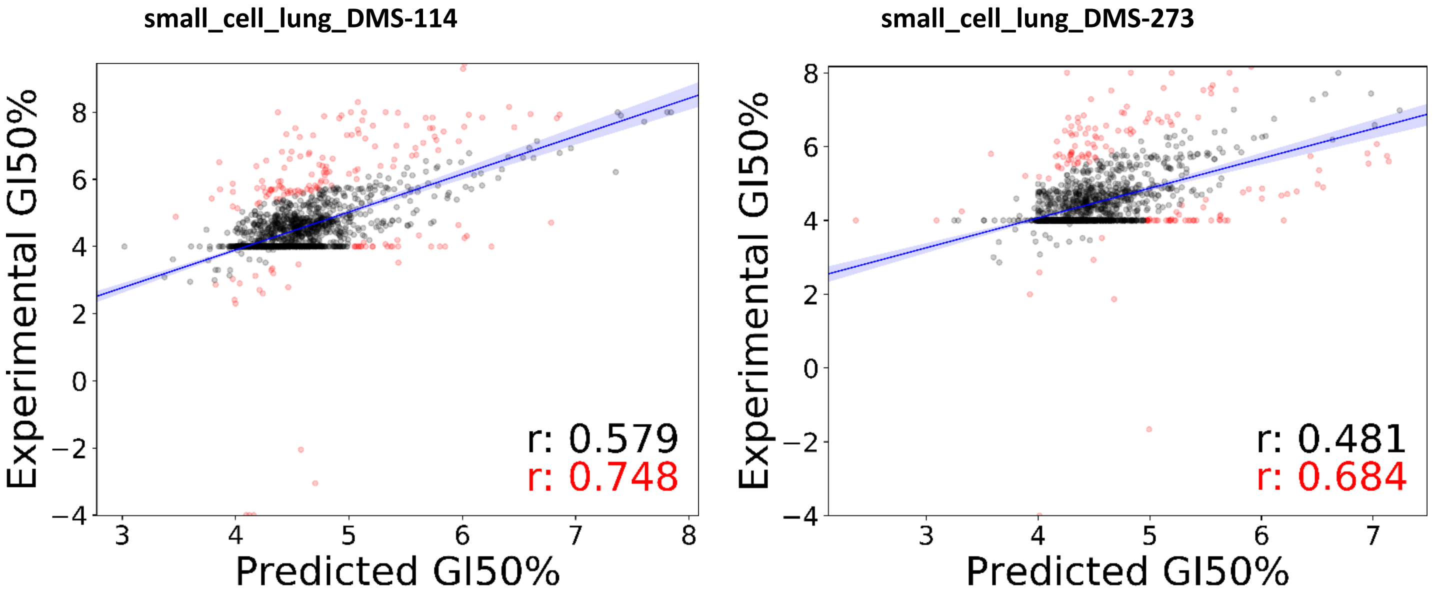

Figure S21. Performance of pdCSM-cancer (Small cell lung cancer panel) on independent blind tests. Scatter plots between experimental and predicted GI50% values given in -log 10(molar) for each of the cell line models of the small cell lung panel are displayed. Pearson's correlation coefficient (r) is shown for each scatter plot (in black for 100% of the data and red for 90% of the data, after 10% outlier removal).

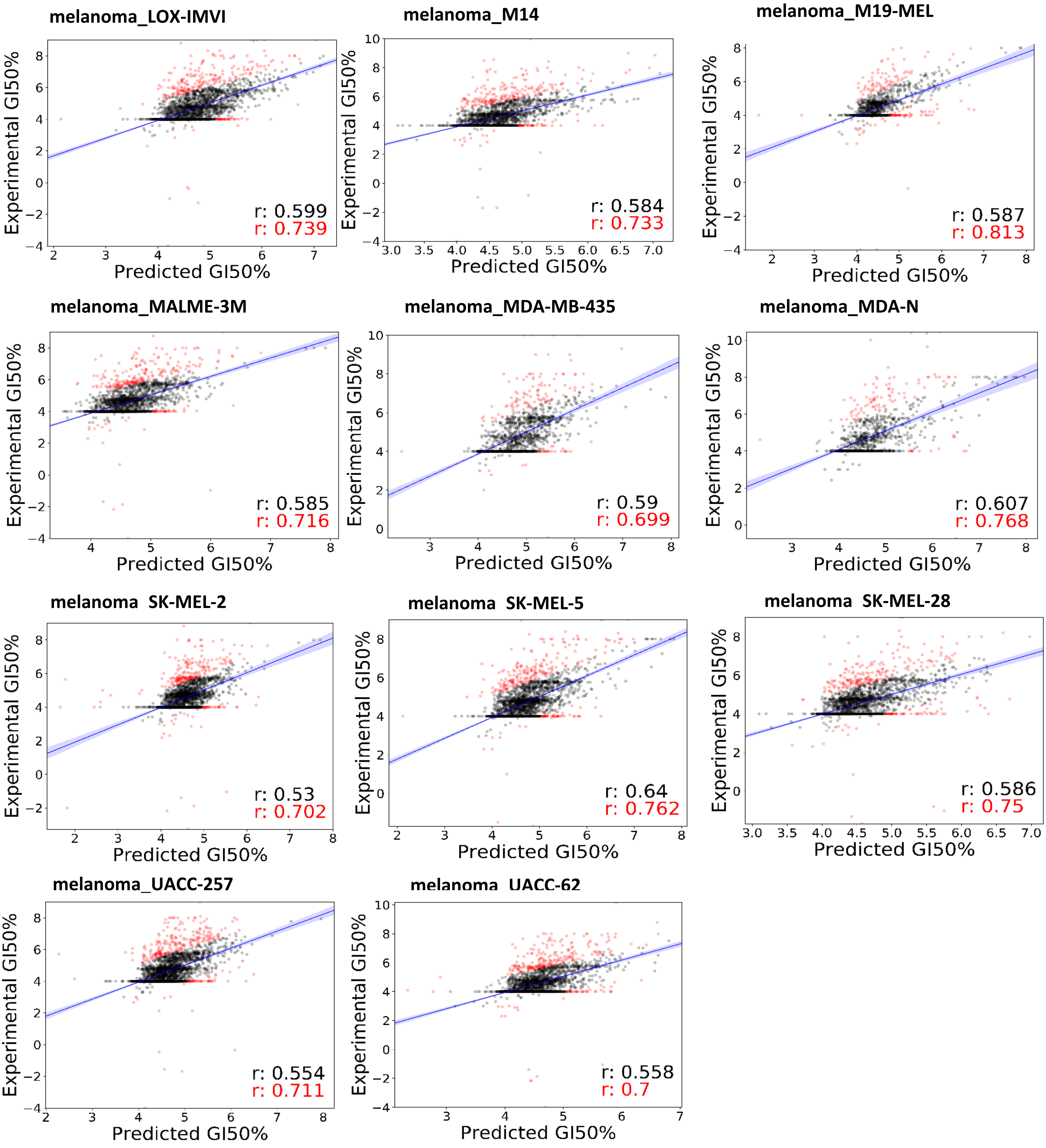

Figure S22. Performance of pdCSM-cancer (Melanoma panel) on independent blind tests. Scatter plots between experimental and predicted GI50% values given in -log 10(molar) for each of the cell line models of the Melanoma panel are displayed. Pearson's correlation coefficient (r) is shown for each scatter plot (in black for 100% of the data and red for 90% of the data, after 10% outlier removal).

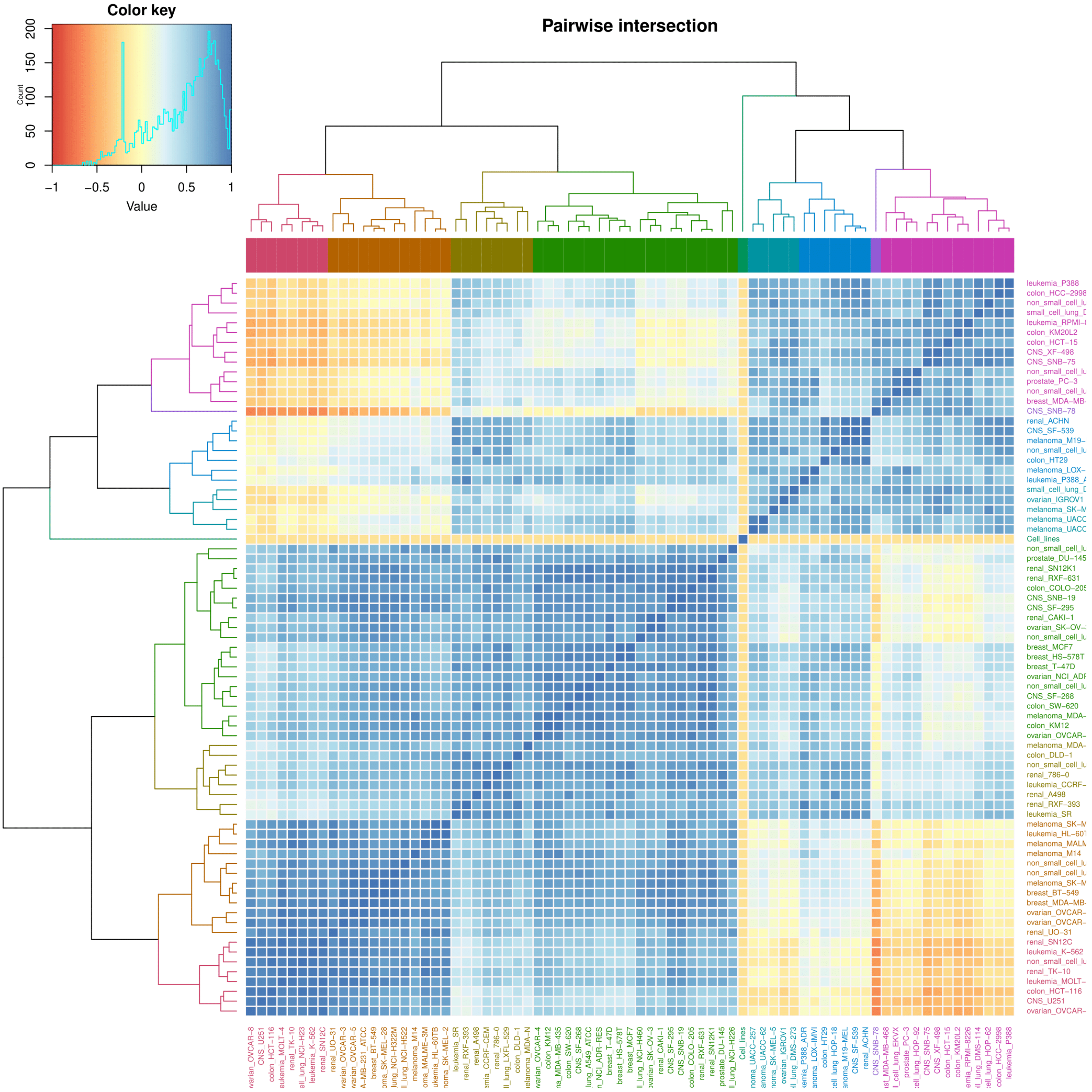

Figure S23. Pairwise intersection of common molecular properties in the NCI60 cancer cell lines (panels). The heatmap displays the pairwise intersection between the common features of the molecules, in different cancer cell lines/panels, as well as their hierarchical clustering. Cell lines per tissue type do not cluster together, apart from a small group of Melanoma cell lines, which tend to form a small cluster.

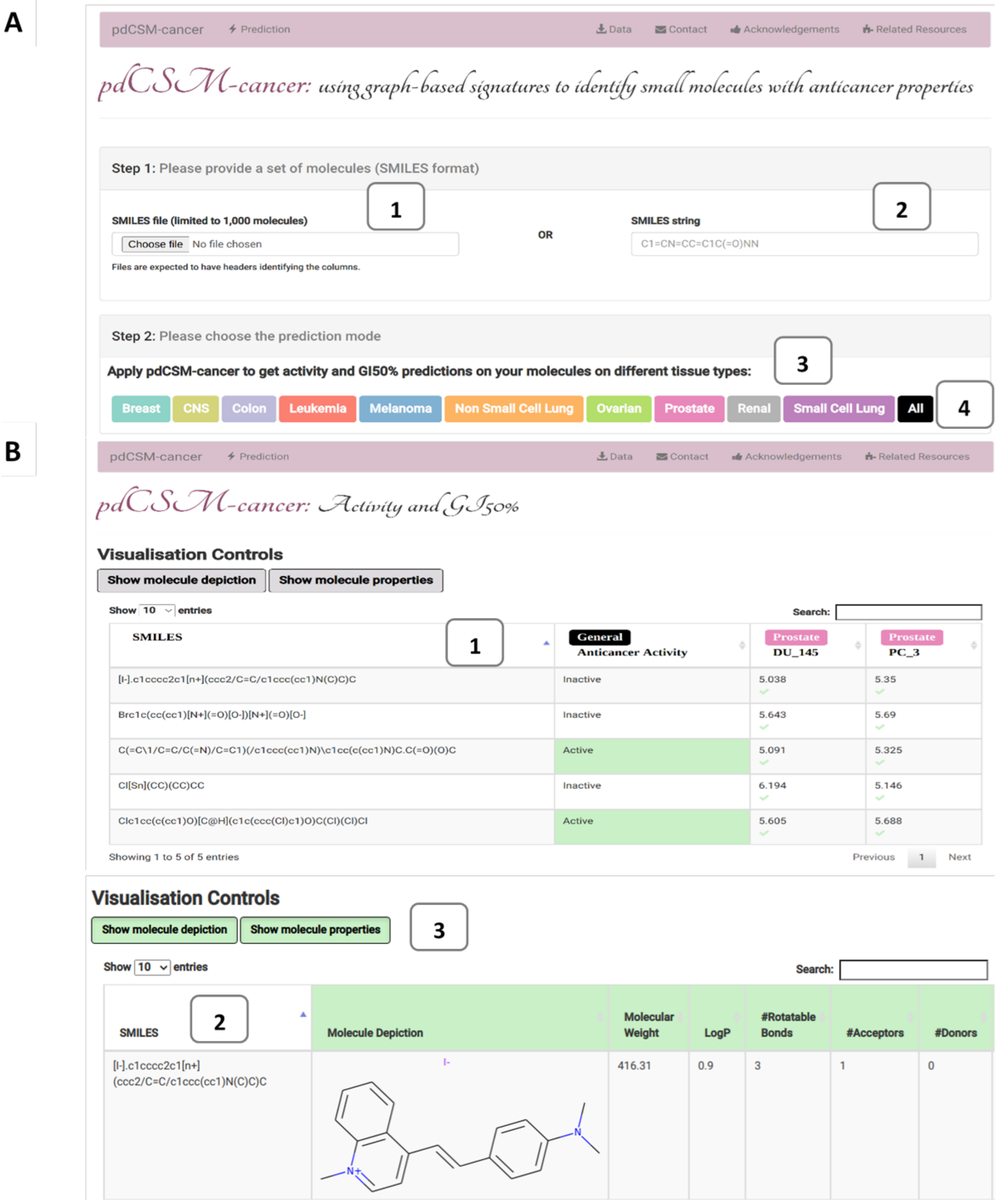

Figure S24. pdCSM-cancer Web Server. Users are directed to the submission page by clicking on "Prediction" at the top menu. (A) At the submission page users can either submit a set of compounds as a SMILES file (1) or an individual compound as a SMILES string (2). Users have the options to either choose different prediction modes according to their tissue of interest (3) or they can choose to run all tissues (4). After choosing the prediction mode of interest, users will be redirected to a results page (B) where predictions for all 74 cancer cell lines (9 tissue types) specific models, anticancer activity (GI50%), general anticancer model (1), physiochemical properties and molecular depiction are presented in tabular format (2). Users have the options to either show or hide the molecule properties and depiction (3).

Graph based-signatures

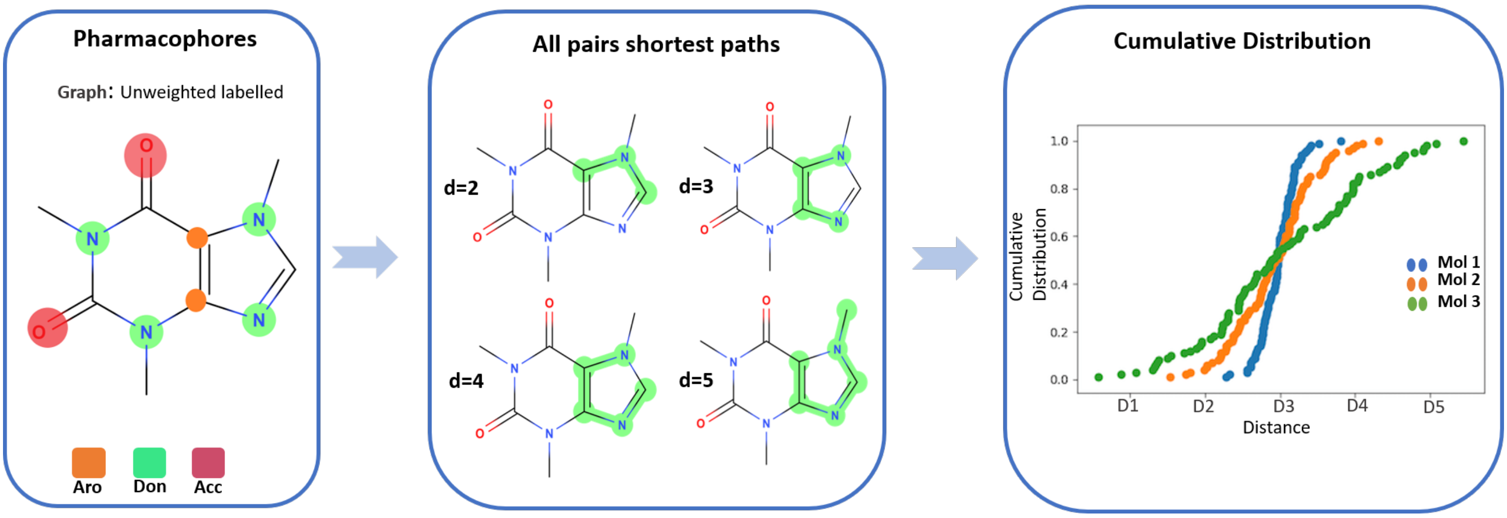

Figure S25. Small molecule bioactivity modelling using graph-based signatures. Small molecules are represented as unweighted graphs in which atoms are represented as nodes and their chemical bonds as edges. Atoms are labelled by Pharmacophore modelling (left panel). All-pairs shortest paths are computed as a distance between any two nodes of the molecular graph (middle panel) to express the geometry and physicochemical properties of the small molecules as cumulative distributions (right panel).

TABLES

Table S1. Complementary features applied in pdCSM-cancer calculated using the RDKit tool.

Name of Features	Descriptions
**General physiochemical properties**	
TPSA	Topological polar surface area (TPSA)
NumRotatableBonds	Number of Rotatable Bonds
MolWt	Molecular Weight
MolLogP	Molecular logP
Fcount	Number of Fluorine atoms
RingCount	Number of rings
NumHDonors	Number of H-bond donors
NumHAcceptors	Number of H-bond acceptors
**Descriptors of Surface area**	
SlogP_VSA1-12	MOE-type, LogP and surface area contributions
EState_VSA1-11	MOE-type, EState indices and surface area contributions
PEOE_VSA1 - 14	MOE-type, partial charges and surface area contributions
SMR_VSA1 - 10	MOE-type, surface area and molar refractivity contributions
**Graph-based indices**	
BertzCT	Topological index of the complexity of molecules

Kappa1-3	Index of the molecular shape
BalabanJ	Balaban's topological index
Chi0n-4n	Index of Molecular connectivity

Table S2. Performance of pdCSM-cancer using different evaluation metrics. Values correspond to the performance of the final models as Pearson's correlation, RMSE and Kendall under 10-fold cross validation. "RF and ET correspond to Random Forest and Extremely Randomised Trees algorithms."

Tissues	Cell Lines	Algorithm	Pearson (100%)	Pearson (90%)	RMSE (100%)	RMSE (90%)	Kendall (100%)	Kendall (90%)
**CNS**	**SF-268**	**ET**	**0.666**	**0.811**	**0.622**	**0.371**	**0.487**	**0.536**
	**SF-295**	**ET**	**0.681**	**0.821**	**0.604**	**0.367**	**0.489**	**0.533**
	**SF-539**	**ET**	**0.666**	**0.812**	**0.636**	**0.393**	**0.492**	**0.548**
	**SNB-19**	**RF**	**0.634**	**0.774**	**0.611**	**0.343**	**0.469**	**0.502**
	**SNB-75**	**RF**	**0.609**	**0.75**	**0.657**	**0.407**	**0.448**	**0.502**
	**SNB-78**	**RF**	**0.578**	**0.739**	**0.601**	**0.305**	**0.396**	**0.415**
	**U251**	**ET**	**0.69**	**0.818**	**0.615**	**0.373**	**0.506**	**0.55**
	**XF-498**	**ET**	**0.602**	**0.81**	**0.722**	**0.385**	**0.415**	**0.465**
**Breast**	**BT-549**	**ET**	**0.65**	**0.793**	**0.719**	**0.46**	**0.499**	**0.574**
	**HS-578T**	**RF**	**0.635**	**0.763**	**0.738**	**0.473**	**0.472**	**0.535**
	**MCF7**	**ET**	**0.696**	**0.811**	**0.775**	**0.536**	**0.521**	**0.588**

	**MDA-MB-231_ATCC**	**ET**	**0.676**	**0.812**	**0.698**	**0.448**	**0.519**	**0.593**
	**MDA-MB-468**	**ET**	**0.581**	**0.738**	**0.772**	**0.532**	**0.378**	**0.445**
	**T-47D**	**ET**	**0.641**	**0.799**	**0.731**	**0.476**	**0.496**	**0.583**
**Colon**	**COLO-205**	**ET**	**0.669**	**0.826**	**0.659**	**0.4**	**0.496**	**0.552**
	**DLD-1**	**RF**	**0.642**	**0.806**	**0.63**	**0.337**	**0.444**	**0.474**
	**HCC-2998**	**ET**	**0.631**	**0.792**	**0.633**	**0.389**	**0.465**	**0.519**
	**HCT-116**	**ET**	**0.697**	**0.823**	**0.654**	**0.415**	**0.515**	**0.565**
	**HCT-15**	**ET**	**0.631**	**0.784**	**0.669**	**0.417**	**0.474**	**0.524**
	**HT29**	**ET**	**0.674**	**0.815**	**0.639**	**0.389**	**0.494**	**0.547**
	**KM12**	**ET**	**0.664**	**0.809**	**0.612**	**0.373**	**0.493**	**0.544**
	**KM20L2**	**ET**	**0.621**	**0.836**	**0.638**	**0.315**	**0.435**	**0.47**
	**SW-620**	**RF**	**0.656**	**0.791**	**0.677**	**0.428**	**0.475**	**0.526**
**Leukaemia**	**CCRF-CEM**	**ET**	**0.652**	**0.797**	**0.733**	**0.479**	**0.482**	**0.542**
	**HL-60TB**	**ET**	**0.636**	**0.793**	**0.748**	**0.484**	**0.477**	**0.549**
	**K-562**	**ET**	**0.663**	**0.824**	**0.703**	**0.439**	**0.495**	**0.561**
	**MOLT-4**	**ET**	**0.671**	**0.824**	**0.696**	**0.444**	**0.492**	**0.566**
	**P388_ADR**	**ET**	**0.696**	**0.806**	**0.994**	**0.698**	**0.493**	**0.563**
	**P388**	**ET**	**0.741**	**0.856**	**1.035**	**0.731**	**0.532**	**0.605**

	RPMI-8226	ET	0.636	0.789	0.715	0.452	0.48	0.547
	SR	ET	0.631	0.783	0.819	0.529	0.477	0.539
Ovarian	IGROV1	ET	0.658	0.807	0.629	0.383	0.479	0.526
	NCI_ADR-RES	ET	0.654	0.777	0.714	0.466	0.498	0.555
	OVCAR-3	ET	0.669	0.807	0.644	0.399	0.499	0.554
	OVCAR-4	ET	0.634	0.808	0.611	0.363	0.49	0.554
	OVCAR-5	ET	0.641	0.807	0.567	0.322	0.475	0.512
	OVCAR-8	ET	0.682	0.824	0.606	0.366	0.502	0.552
	SK-OV-3	ET	0.654	0.794	0.568	0.337	0.452	0.49
Prostate	DU-145	ET	0.675	0.813	0.719	0.466	0.508	0.577
	PC-3	ET	0.685	0.818	0.731	0.468	0.516	0.587
Renal	786-0	ET	0.651	0.807	0.643	0.386	0.49	0.54
	A498	ET	0.635	0.786	0.627	0.375	0.469	0.516
	ACHN	RF	0.644	0.77	0.652	0.399	0.474	0.513
	CAKI-1	ET	0.653	0.817	0.671	0.408	0.495	0.551
	RXF-393	ET	0.648	0.803	0.663	0.412	0.496	0.558
	RXF-631	ET	0.655	0.822	0.588	0.291	0.432	0.463
	SN12C	ET	0.651	0.817	0.621	0.36	0.486	0.534

	SN12K1	ET	0.743	0.848	0.953	0.647	0.535	0.595
	TK-10	ET	0.637	0.791	0.567	0.335	0.471	0.51
	UO-31	ET	0.649	0.799	0.624	0.389	0.486	0.542
Non small-cell Lung	A549_ATCC	ET	0.676	0.822	0.593	0.349	0.486	0.532
	EKVX	RF	0.59	0.737	0.599	0.352	0.456	0.493
	HOP-18	ET	0.536	0.774	0.662	0.338	0.385	0.436
	HOP-62	ET	0.646	0.807	0.622	0.356	0.474	0.518
	HOP-92	ET	0.6	0.764	0.694	0.425	0.465	0.529
	LXFL-529	ET	0.667	0.832	0.601	0.33	0.452	0.491
	NCI-H226	ET	0.635	0.801	0.616	0.36	0.471	0.52
	NCI-H23	ET	0.694	0.816	0.602	0.373	0.504	0.548
	NCI-H322M	ET	0.639	0.801	0.574	0.323	0.471	0.502
	NCI-H460	ET	0.694	0.833	0.658	0.397	0.494	0.537
	NCI-H522	ET	0.666	0.807	0.704	0.451	0.501	0.563
Small-cell Lung	DMS-114	ET	0.674	0.831	0.616	0.4	0.462	0.467
	DMS-273	ET	0.579	0.837	0.742	0.346	0.402	0.507
	LOX-IMVI	ET	0.665	0.809	0.69	0.435	0.491	0.549
	M14	ET	0.655	0.815	0.655	0.399	0.486	0.541
	M19-MEL	ET	0.656	0.83	0.604	0.319	0.466	0.488

**Melanoma**	**MALME-3M**	**ET**	**0.644**	**0.802**	**0.631**	**0.384**	**0.479**	**0.532**
	**MDA-MB-435**	**ET**	**0.682**	**0.817**	**0.762**	**0.508**	**0.505**	**0.588**
	**MDA-N**	**ET**	**0.658**	**0.783**	**0.731**	**0.434**	**0.466**	**0.513**
	**SK-MEL-28**	**ET**	**0.658**	**0.802**	**0.56**	**0.337**	**0.48**	**0.518**
	**SK-MEL-2**	**RF**	**0.615**	**0.755**	**0.634**	**0.379**	**0.463**	**0.503**
	**SK-MEL-5**	**ET**	**0.657**	**0.813**	**0.644**	**0.4**	**0.501**	**0.565**
	**UACC-257**	**RF**	**0.622**	**0.761**	**0.611**	**0.372**	**0.468**	**0.511**
	**UACC-62**	**RF**	**0.638**	**0.773**	**0.667**	**0.417**	**0.469**	**0.517**
